# GPCR-Based Dopamine Sensors—A Detailed Guide to Inform Sensor Choice for In Vivo Imaging

**DOI:** 10.3390/ijms21218048

**Published:** 2020-10-28

**Authors:** Marie A. Labouesse, Reto B. Cola, Tommaso Patriarchi

**Affiliations:** 1 Department of Psychiatry, College of Physicians and Surgeons, Columbia University, New York, NY 10032, USA; mal2307@cumc.columbia.edu; 2 Division of Molecular Therapeutics, New York State Psychiatric Institute, New York, NY 10032, USA; 3 Anatomy and Program in Neuroscience, University of Fribourg, 1700 Fribourg, Switzerland; reto.cola@uzh.ch; 4 Institute of Pharmacology and Toxicology, University of Zurich, 8057 Zurich, Switzerland; 5 Neuroscience Center Zurich, University and ETH Zurich, 8057 Zurich, Switzerland

**Keywords:** behavior, drug screening, genetically encoded, dopamine, fiber photometry, fluorescent biosensor, in vivo fluorescent imaging, neuromodulator, pharmacology

## Abstract

Understanding how dopamine (DA) encodes behavior depends on technologies that can reliably monitor DA release in freely-behaving animals. Recently, red and green genetically encoded sensors for DA (dLight, GRAB-DA) were developed and now provide the ability to track release dynamics at a subsecond resolution, with submicromolar affinity and high molecular specificity. Combined with rapid developments in in vivo imaging, these sensors have the potential to transform the field of DA sensing and DA-based drug discovery. When implementing these tools in the laboratory, it is important to consider there is not a ‘one-size-fits-all’ sensor. Sensor properties, most importantly their affinity and dynamic range, must be carefully chosen to match local DA levels. Molecular specificity, sensor kinetics, spectral properties, brightness, sensor scaffold and pharmacology can further influence sensor choice depending on the experimental question. In this review, we use DA as an example; we briefly summarize old and new techniques to monitor DA release, including DA biosensors. We then outline a map of DA heterogeneity across the brain and provide a guide for optimal sensor choice and implementation based on local DA levels and other experimental parameters. Altogether this review should act as a tool to guide DA sensor choice for end-users.

## 1. Introduction

### 1.1. Measuring Neuromodulator Release During Behavior

Animals must constantly adjust their behavior to meet the demands of ever-changing sensory inputs, external environments and internal needs. Neuromodulators, such as dopamine (DA), provide one evolutionary conserved mechanism that supports this behavioral adaptability. By rapidly modifying the properties of their target neurons, neuromodulators can deeply affect neural circuits and in turn modulate behavior [1,2,3,4]. Disturbances in neuromodulatory signaling pathways are associated with a large number of behavioral dysfunctions and brain pathologies including psychotic and mood disorders, motor diseases or addiction. A key challenge for neuroscientists is the ability to understand how neuromodulators encode and control behavioral outputs in health and disease states and in turn how these neuromodulators can be harnessed to treat brain disorders. The ability to answer these questions is dependent on available technologies that can reliably monitor neuromodulatory processes, including (i) action potential (AP) propagation and (ii) synaptic release. Advanced in vivo imaging methods to track AP propagation in genetically defined cells have been developed across the past decades, most notably in vivo calcium imaging, the method of choice for monitoring intracellular calcium levels as a proxy for AP propagation [5]. Calcium imaging relies on the usage of high resolution genetically encoded calcium indicators (GECIs) (e.g., GCaMP), which detect calcium-dependent changes in the chromophore environment of ultrasensitive circularly permuted fluorescent proteins (cpFP) (e.g., the green cpGFP) [6,7,8,9]. 

However, neuromodulator release does not linearly correlate with AP propagation but instead can undergo local regulation in an AP-independent manner, for example via presynaptic autoreceptor mechanisms [10,11]. Once released, neuromodulators act onto their cognate receptors expressed on the membranes of receiving cells. Receptor activation modulates downstream signaling cascades which in turn can dramatically impact vesicular release probability, firing patterns, excitability or plasticity within the local microcircuit [2,3,4,12]. Because neuromodulator kinetics are selectively regulated by release and reuptake mechanisms, the time they spend in the extracellular space directly relates to their downstream actions [13]. Thus, the ability to measure extracellular levels of neuromodulators with high spatiotemporal resolution during behavior becomes essential to gain deeper insights into how neuromodulator release encodes behavior. 

### 1.2. Heterogeneity of Brain Dopamine Systems

Dopamine is one of several neuromodulators broadly expressed throughout the brain [14]. The DA system is best known for its roles in reward behavior [15,16,17,18], action learning [19] and motor function [20] but its effects extend to many other functional domains. For instance, DA has been extensively shown to regulate cognitive function [21,22], aversive processing [17,23], social interaction [24], feeding behavior [25,26,27,28], physical activity [29,30,31] or metabolic and hormonal homeostasis [32,33,34]. These many functions are modulated by a broad network of DA projection neurons, arising from nine major DAergic cell groups labeled A8 to A16 [35], as originally introduced by Dahlström and Fuxe in 1964 [36]. Thus, while DA release from ventral midbrain neurons into the dorsal striatum and nucleus accumbens (NAc) are by far the most studied [14], DA is also released in a sparser fashion by neurons with cell bodies in the hypothalamus [37], dorsal raphe [24,38] or locus coeruleus [39,40], to name a few. Moreover, responses to DA can be found in many DA-recipient regions including the medial prefrontal cortex (mPFC) [22,23], hippocampus [24], midbrain [41], paraventricular thalamus (PVT) [39], amygdala [24], septum [42] or ventral pallidum [43] and globus pallidus (GPe) [44]. Importantly, there is a large regional heterogeneity in DA innervation patterns and DA concentrations across brain regions. While the dorsal striatum and NAc are heavily innervated by dense DA projections arising from the midbrain, other brain regions receive much sparser projections [35,45,46]. Moreover, basal and evoked DA levels measured by analytical methods can also vary by a factor of at least 10 in more sparsely innervated regions as compared to the striatum [47]. For example, Koch et al., (2002) reported basal DA levels of 5.8 ± 0.7 nM in the dorsal striatum, 4.5 ± 1.6 nM in the NAc, 0.26 ± 0.05 nM in the hypothalamus and 0.30 ± 0.1 nM in the mPFC in awake rats using microdialysis [48], showing the large variability across brain regions.

### 1.3. Measuring Dopamine Across Brain Regions

Thus, a key challenge for systems and behavioral neuroscientists is the ability to measure DA across densely and sparsely innervated regions and to correlate in vivo DA levels with relevant behavioral outputs. The ability to answer these questions is dependent on available technologies that can reliably monitor DAergic processes in the slice or the awake behaving animal, even when DA concentrations are low. Historically, in vivo measurements for DA have been tackled with classical analytical chemistry techniques, such as microdialysis or fast-scan cyclic voltammetry (FSCV). These methods have transformed our ability to measure neuromodulators in vivo in the past five decades but face limitations that prevent long-term measurements at adequate spatiotemporal resolution and/or specificity, in particular in regions with relative sparse DA innervation. In the past few years, however, scientists have developed a large palette of genetically encoded sensors that surpass some of these technical barriers. This initially included cell-based sensors that are based on dual wavelength FRET imaging (CNiFERs) [49] and other probes that track DA receptor-dependent changes in gene expression (iTango2 and SPARK) [50,51]. Genetically encoded calcium sensors, in particular those targeted to the axon or synapse (axon-GCAMP, synaptophysin-GCAMP) [52,53] also provide indirect measures of DA release by virtue of measuring axonal and/or presynaptic calcium transients in DA neurons and can track activity even if the local DA release levels are low [23,54,55]. Technologies that track DA release in brain slices have also been developed in recent years, most notably in the form of false fluorescent neurotransmitters (FFNs) [56] and carbon nanotubes [57]. Although they are more difficult to apply in vivo, they provide useful ex vivo platforms to study DA dynamics with high spatiotemporal resolution and sensitivity.

**Figure 1:**
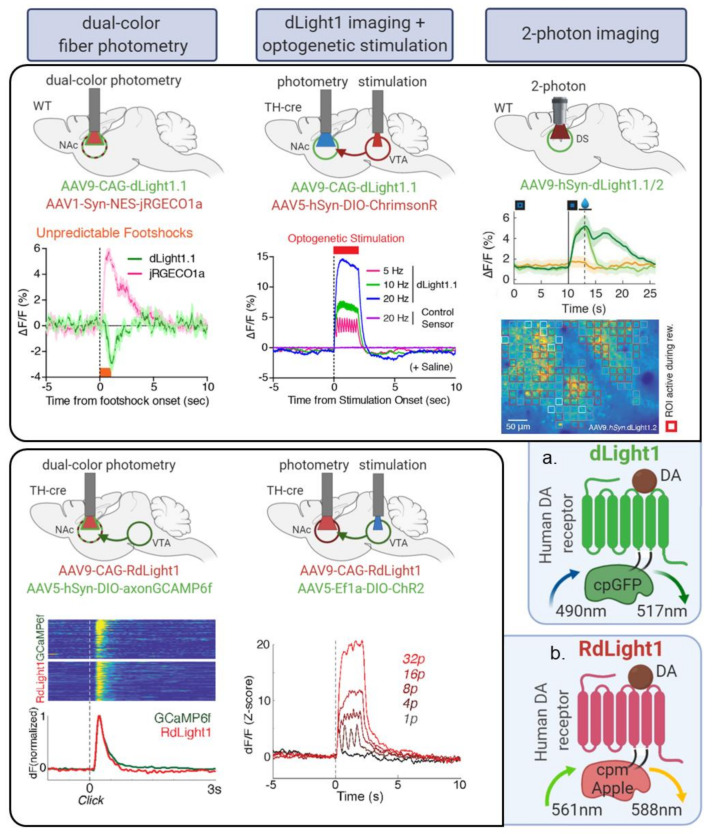
In vivo applications of dLight1 and RdLight1 dopamine (DA) sensors in mice. Graphs show normalized fluorescent responses (dFF). (**a**) dLight1 variants were validated in multiple imaging and experimental modalities: (i) dual-color fiber photometry of nucleus accumbens (NAc) cells using the red-shifted calcium sensor jRGECO1a [77] and local DA release using dLight1.1 following unpredictable shock exposure (dotted line); (ii) fiber photometry imaging of DA release using dLight1.1 in the NAc following optogenetic stimulation (5–20 Hz, 2 seconds) of DA cell bodies in the ventral tegmental area (VTA) using the red-shifted opsin ChrimsonR; (iii) two-photon imaging of DA release (dLight1.2) (top) across heterogeneous sites in motor cortex (M1/M2) across 17 μM large regions of interest (= red square ROIs, bottom), here showing ROIs responsive to locomotion/reward expectation vs. rest (green vs. orange) at the Go cue (vertical line) and increases in fluorescence upon reward delivery (dark green) but not reward omission (light green) (population data) (middle). (**b**) RdLight1 validations: (i) dual-color fiber photometry of VTA terminals in the NAc using the green axon-targeted calcium sensor axon-GCaMP6f [52] and local DA release using RdLight1 following unexpected, audible reward deliveries (dotted line) and (ii) fiber photometry imaging of DA release using RdLight1 in the NAc following optogenetic stimulation (1–32 pulses; 2 seconds) of DA cell bodies in the VTA using the green opsin ChR2.

More recently, we and others developed red and green genetically encoded DA biosensors based on cpFPs (previously used for calcium sensors) engineered into a DA receptor, which now allow us to track fluorescent changes at a subsecond resolution, with submicromolar affinity and high molecular specificity. These include our 8 red, yellow and green RdLight1/YdLight1/dLight1sensors [58,59], as well as the 6 GRAB-DA sensors, all harboring their own specific properties [60,61]. Combined with rapid developments in in vivo imaging techniques [62], such as multiplexed fiber photometry [63,64,65], head-mounted microendoscopes [66,67] and 2-photon endoscopy and mesoscopy [68,69], these sensors have the potential to transform the field of DA sensing as they allow us to monitor DA dynamics in vivo with unprecedented spatiotemporal resolution (see Figure 1 for examples of applications). 

### 1.4. Sensor Choice Depends on Brain Region Dopamine Levels and Other Experimental Modalities

When implementing these tools in a new brain region, it is important to keep in mind that often there is not a ‘one-size-fits-all’ sensor. Sensor properties, most importantly their ligand-binding affinity must be carefully chosen to match local levels of DA in the brain region of interest (ROI). Moreover, experimental paradigms largely differ from each other where, for each given question, specific sensor properties take priority. For instance, fast sensor kinetics are most crucial in behavioral experiments harboring highly frequent stimuli and events but sensor molecular specificity will matter most when attempting to distinguish the dual dynamics of two neuromodulators (e.g., DA/noradrenaline, NE). Sensor spectrum will be important in multiplex experiments and sensor brightness will be key in 1 and 2-photon imaging. Importantly, G-protein coupled receptor (GPCR) sensors inherit the pharmacological properties of their parent DA receptor [58,59], making sensor fluorescence a putative readout for receptor subtype activation: this feature may be incompatible with certain pharmacological applications but can also be harnessed as a tool to screen candidate drugs based on their ability to engage certain receptor subtypes.

In this review, we start by briefly reviewing the broad number of techniques to monitor DA release (for further review see Reference [70]), including the recently developed genetically encoded DA sensors. We then outline a detailed map of DA innervation patterns as well as basal and evoked DA levels across the brain. We use this map to illustrate how end-users can optimize sensor choice according to their brain ROI. We also provide a guide for sensor choice based on other experimental parameters. We primarily focus on in vivo imaging conditions in rodents but this review can also inform research in other animal models or in vitro assays. Moreover, these guidelines are likely to be relevant for other existing neuromodulator sensors such as NE, acetylcholine, serotonin or adenosine sensors [71,72,73,74,75,76].

## 2. Currently Available Methods to Measure Dopamine Release

### 2.1. Analytical Chemistry: Microdialysis (In Vivo) and FSCV (Ex Vivo and In Vivo)

Analytical chemistry techniques such as microdialysis, FSCV and amperometry have provided critical tools to measure brain levels of DA ex vivo and in vivo across the past five decades [78]. These methods have transformed our understanding of DA function and their relations to behavior. Microdialysis is an in vivo technique based on dialysate sampling of cerebrospinal fluid through a semi-permeable membrane-containing probe implanted into the brain tissue of a living animal. Samples are analyzed by analytical techniques such as high-performance liquid chromatography (HPLC). Microdialysis has excellent sensitivity (nanomolar) and molecular specificity and can therefore discriminate amongst a plethora of different neuromodulators including DA, NE, serotonin, acetylcholine and others. Thus, microdialysis remains an essential quantitative tool to evaluate the basal concentration of multiple neurotransmitters simultaneously in identified brain regions. However, due to constraints on dialysate dilution, neurotransmitter diffusion kinetics and slow collection times, the temporal resolution of microdialysis is slow: on the scale of minutes (five minutes on average). The spatial resolution is also low, for example, ~0.5 mm for a 200 μm diameter microprobe [79,80]. As a consequence, microdialysis is limited in its ability to track phasic changes in DA release or transients of small magnitude in regions of sparse innervation as these fast or small changes are diluted within the dialysate over time. Thus, although microdialysis has been successfully used in regions with sparse DA like the mPFC to track basal and evoked DA, they do not succeed in capturing the full temporal dynamics of release due to these limitations. For instance, low levels of DA near detection threshold could be reported in the GPe in vivo but this was only achieved by accruing the dialysate over tens of minutes, thus losing crucial temporal information [81].

Electrochemical methods such as FSCV use a carbon-fiber probe inserted into the brain (in vivo) or onto a slice (ex vivo) to detect voltage-dependent oxidation/reduction profiles characteristic of specific neurotransmitters [82]. FSCV offers excellent sensitivity (nanomolar) and high temporal resolution (subsecond range). The thin (7 μm) implanted carbon fiber electrodes cause minimal intracerebral damage, offers superior spatial resolution (< 10 μm) vs. microdialysis [82] and in principle microarrays can be used to record multiple channels at once [83,84]. FSCV thus remains an essential tool to estimate the evoked concentration of DA in identified brain regions in a quantitative manner, including in brain regions with sparse DA innervation. FSCV does, however, rely on background subtraction and lacks the molecular specificity necessary to discriminate neuromodulatory molecules from their metabolites which have similar electrochemical properties [82]. For instance, FSCV does not distinguish DA from NE, an important limitation for brain regions with dual innervation like the mPFC [85]. However, FSCV is not affected by the presence of DA receptor ligands (as is the case for dLight and GRAB-DA, see Section 5.6), making FSCV a useful method to deploy in combination with drug treatments and/or in drug addiction research.

Finally, it is worth noting that analytical chemistry methods are also not genetically encoded and therefore lack the cellular resolution required for many applications.

### 2.2. False Fluorescent Neurotransmitters (Ex Vivo)

False fluorescent neurotransmitters (FFNs) represent another highly relevant technique to monitor DA release, primarily in brain slices. FFNs are hydrophilic fluorescent probes that mimic the topology and properties of the neurotransmitter of interest and can therefore be taken up into synaptic vesicles and discharged during exocytosis; thus tracking FFN staining and destaining in the vesicle provides an indirect measure of synaptic release dynamics. For instance, FFN200 is a substrate for the vesicular monoamine transporter, VMAT2, that has been specifically engineered to track DA or NE release in slices. One main advantage of FFNs, in particular FFN200, is the ability to track synaptic vesicle fusion and exocytosis from individual single-site synaptic release sites on the axon, thus granting exceptional spatial resolution [56,86]. Moreover, pH-sensitive FFNs such as FFN102 were also developed to increase FFN signal to noise ratio (SNR) by harnessing the existing pH gradient that differentiates acidic synaptic vesicles from the extracellular space. Thus FFN102 is particularly useful to track DA release in brain regions with sparse innervation such as demonstrated in the GPe in Reference [44]. It is important to note, however, that FFN imaging of DA release has hitherto not been applied successfully in vivo due to the difficulty of the in vivo loading process. In vivo imaging of FFN270 for NE has recently been performed using 2-photon imaging through a cranial window, indicating the potential of these techniques to extend to the in vivo preparation [87]. Other limitations for in vivo experiments include the lack of molecular specificity of FFNs for DA over NE and the relatively low temporal resolution (several seconds).

### 2.3. Carbon Nanotubes (Ex Vivo)

Another method to track DA release ex vivo includes single-walled carbon nanotubes (SWCNT) [88]. Carbon nanotubes are cylindrical molecules that consist of rolled-up sheets of single-layer carbon atoms with diameters typically in the range of nanometers. To develop an optical probe for catecholamines, including DA, near-infrared intrinsically fluorescent SWCNTs were pinned with catecholamine-sensitive functional polymers such that the fluorescent signal increases upon binding with catecholamines (DA or NE) [57,88]. When applied to brain slices, newly developed catecholamine SWCNTs (nIRCat) offered excellent temporal resolution (subsecond), comparable to that of FSCV or DA biosensors. Moreover, preliminary results indicated that nIRCat can detect heterogeneity of DA release sites with a spatial resolution in the single-digit micrometer range (4 μM ROIs) even in brain slices [57]. Prior work in cells also showed that a different version of catecholamine SWCNTs could be positioned into nanoarrays, allowing to dissect the spatial spread of DA transmission with unsurpassed spatiotemporal resolution (<1 μM ROIs) along the membrane of single neurons in culture [89] (see also [90] for a detailed review). These nanoarray methods can hopefully be applied to brain slices in the future, possibly even by tailoring their location to for example postsynaptic sites using recognition motifs [90]. Unlike DA biosensors, catecholamine SWCNTs are resistant to photobleaching. They also remain insensitive to DA ligands and are thus more easily combined with DA drug manipulations or drug addiction investigations [57]. Due to their near infrared fluorescent profile, catecholamine SWCNTs are potentially compatible with mesoscopic through-cranium imaging or multiphoton imaging due to low light absorption and scattering and low autofluorescence [90]; although this needs to be determined in future studies. One hurdle is the lack of molecular specificity, as catecholamine SWCNTs are sensitive to both DA and NE [57], which remains problematic in brain regions with dual DA/NE innervation like the mPFC. Moreover, as catecholamine SWCNTs are not genetically encoded and need to be loaded by diffusion [57], it remains to be determined whether they can reliably be deployed into an in vivo preparation.

### 2.4. GPCR FRET-Based Sensors: CNiFERs (Ex Vivo and In Vivo)

Another recent addition to the toolbox to measure DA release in vivo is based on the expression of GPCRs coupled to Gq in clonal cell lines such as HEK293 cells (CNiFERs: cell-based neurotransmitter fluorescent engineered reporters) [49,91]. Upon activation of these GPCRs, the Gq pathway activates phospholipase C and hence results in IP3-mediated release of intracellular calcium from the endoplasmic reticulum. This increase in intracellular calcium is detected by a FRET-based calcium-sensor co-expressed by the clonal cell line. This approach has been successfully used to sense DA and NE in vivo at nanomolar concentrations with reasonable spatial resolution (100 μM) [91]. This was achieved with high molecular specificity given that CNiFERs inherit the molecular specificity of the GPCR they express. One limitation, however, is the low temporal resolution (5 seconds) due to the dependence on Gq downstream signaling. Moreover, the invasive nature of the cell implantation and associated mild inflammation, hinders experiments longer than 1 week post injection [49]. However, due to their higher resistance to photobleaching, CNiFERs can be particularly useful to track DA changes across broad time periods for example, hours-long sessions or across days.

### 2.5. GPCR Signal Transduction Sensors: iTango2 and SPARK (Ex Vivo and In Vivo)

Another GPCR-based system developed recently, iTango2, is an inducible genetically encoded system that was designed to capture signaling cascades downstream of GPCR activation, in particular the DA D2 receptor (DRD2). The Tango system was originally developed by fusing the transcription factor tTA to the *C*-terminal domain of the GPCR of interest (e.g., DRD2) via a peptide sequence (TEV) containing a protease cleavage site [92,93]. Upon DA binding to the receptor and GPCR-activation of the beta-arrestin downstream pathway, the TEV is recruited to the receptor, the TEV sequence cleaved, releasing the transcription factor tTA which can in turn activate transcription of a reporter gene (e.g., a fluorescent protein) via interaction with a tetracycline response element (TRE). This system allowed nanomolar sensitivity detection of DA in a restricted brain region, albeit with low temporal resolution (hours) but with, in principle, a spatial resolution of single cells. However, in the improved version iTango2 [51], as well as in SPARK [50], the protease cleavage is further dependent on the presence of blue light, making it possible to optogenetically activate the system on demand ex vivo or in vivo, leading to spatial restriction as well as higher temporal resolution (minutes). One important limitation for these methods in vivo is that they can only report neuromodulator signaling at a single timepoint and thus cannot be used for real time monitoring of DA release. They do, however, represent important tools to track activation of DRD2 over minutes, hours or even days (as the gene expression changes can last several days). Importantly, iTango2 does not only allow to track but also to manipulate cells involved in a task, by expressing genes under the control of TRE following iTango2 labeling. Thus, iTango2 allows to test the causal role of specific cell types activated by DA during behavior.

### 2.6. Genetically Encoded Calcium Sensors (Ex Vivo and In Vivo)

Another method to access DA dynamics is to make use of calcium sensors (GCaMPs) to monitor neural dynamics of DA axons as a proxy for release. Indeed, GCaMPs are widely used to track calcium dynamics in neurons as a proxy for neural activity with the advantage of high spatiotemporal resolution (up to individual dendrites/axons) and cell specificity [7,8]. GCaMPs detect calcium-dependent changes in the chromophore environment of their ultrasensitive cpGFP [6,7,8,9]. Classically, GCaMP expression is enriched in somatodendritic compartments including dendritic spines where they primarily track post-synaptic calcium transients evoked by afferent activity [6]. However, more recent versions of GCaMPs such as GCaMP7 [8] (and to a lesser extent: GCaMP6 [7]) are more broadly expressed in thin subcellular compartments such as axons and thus reliably track axonal and presynaptic calcium transients along the branches induced by action potentials [8]. These sensors can therefore be used to detect activity of long-range DA axons given that the somatodendritic transients (e.g., in the midbrain) detected by GCaMP will not interfere with axonal signals (e.g., in the striatum) as they are physically removed from each other. They have the added advantage of tracking neural activity in brain regions even if the local DA release levels are low. Thus, GCaMPs represent a useful proxy to estimate DA release dynamics; for example, GCAMP6f imaging has been successfully performed in the striatum to evaluate the dynamics of arising midbrain DA signals during locomotion and reward tasks [54,55,94]. 

Moreover, new versions of GCaMP using targeting motifs to enhance expression to the axon (axon-GCaMP6) [52] or the presynapse (Synaptophysin-GCAMP6) [53] were recently developed and thus represent even more precise methods to track axonal/presynaptic activity as a proxy for DA release. For example, we identified high fidelity between axonal calcium levels arising from the midbrain (measured with axon-GCaMP6f) and DA release (measured with RdLight1) in the NAc in a reward task [59]. Of note, Synaptophysin-GCaMP6 has limited photostability and thus should be reserved for experiments of short duration (a few hundreds of seconds) [52]. These tools can be particularly useful in brain regions with sparse neuromodulator innervation where DA sensors may still lack sensitivity (as outlined more in detail below). 

It is important to note, however, that DA release may occur independent of calcium dynamics as it is regulated locally by multiple presynaptic mechanisms including D2 autoreceptors or cholinergic innervation (for reviews on DA release see: [11,18,95,96]), making DA biosensors particularly important in this context. Moreover, several studies have found that somatodendritic DA release in the midbrain is less dependent on calcium as compared to axonal release in the striatum, making GCaMPs a less appropriate tool to study such somatodendritic release mechanisms. Nonetheless, combined monitoring of calcium transients (GCaMPs) and DA release (dLight/GRAB-DA) can represent a powerful platform to understand how neural activity shapes release dynamics, as demonstrated in recent preprints for acetylcholine [97] or DA [98].

## 3. Catalogue of GPCR Biosensors for Dopamine

### 3.1. The GPCR Dopamine Sensor Toolbox (Ex Vivo and In Vivo)

Single-wavelength GPCR biosensors for DA represent the latest method to track DA release ex vivo and in vivo with high spatiotemporal resolution and molecular specificity [99]. For simplicity we now refer to these as DA biosensors. Since GPCRs are native target receptors for many neuromodulators, including DA, we and others recently established an approach to design sensors based on protein engineering of the cognate receptors [58,60,71,72,73]. Sensors were based on the insertion of a green (cpGFP) or red (cpmApple) module (the same ones previously used for GCaMPs or jRGECO1a [6,7,8,77]) into the third intracellular loop of one of the five receptors (= GPCR) for DA (DRD1-DRD5), thus allowing to transform receptor activation into a fluorescent readout. Upon binding of the endogenous ligand (DA or other neuromodulator), the GPCR undergoes a large conformational change leading to an increase in the fluorescent intensity of the cpGFP/cpmApple due to changes in its chromophore environment [6]. Biosensors for DA (dLight1, RdLight1, GRAB-DA1) [58,59,60,100], as well as for other neuromodulators (NE, acetylcholine, adenosine) [71,72,73,74,76] have already been established, while more advanced versions of DA sensors (GRAB-DA2, GRAB-rDA1) [61] and other sensors (serotonin) [75] are in development.

In Table 1 you will find a summary of currently available DA biosensors as well as their main properties, which are further detailed in Section 5. DA biosensors have already generated key findings in the basic understanding of reward behavior [101,102,103,104,105,106,107], thirst regulation [108], feeding behavior [109], addiction [38,110,111], aversive learning [112], depressive-like behavior [98], sleep-wake cycle [113] or to dissect neuromodulator mechanisms in disease models [114,115] using a variety of in vivo imaging modalities shown in Figure 1. DA biosensors can also be used to understand DA release dynamics in vitro or ex vivo [58,59,60,61], as shown for example in Reference [116] where dLight1 was used to understand the metabolic demands and bioenergetic roles of the mitochondria in governing phasic DA release.

### 3.2. Advantages of GPCR Dopamine Biosensors

What are some reasons for the in vivo performance of DA biosensors compared to other available methods? 

First, DA biosensors demonstrated excellent fluorescent sensitivity and brightness in response to ligand binding, making them ideally suited for in vivo imaging. This is because sensors were generated using a cpGFP/cpmApple module that had already been optimized for calcium sensors through iterative processes [6,7,8,77]. This led to the creation of first-generation single-wavelength sensors that already harbor large fluorescence changes during GPCR activation and decent brightness. First- and second-generation GPCR DA biosensors thus demonstrated a peak increase in fluorescence (dFF) of at least 90% and up to 900% in response to ligand binding (see Table 1) (e.g., 340% for both dLight1.2 and GRAB-DA2m), compared to 5% for FRET sensors [117]. These properties have made DA biosensors easy to implement in the laboratory.

Second, GPCR DA sensors have demonstrated a high specificity and affinity for their endogenous ligands, as well as rapid (tens of milliseconds) conformational change following receptor activation. This is because DA biosensors largely inherited the intrinsic properties of their native receptors, including affinity within physiological ranges (e.g., 765 nM for dLight1.2, 90 nM for GRAB-DA2m), molecular specificity (e.g., 70-fold specificity for DA over NE for dLight1.2, 22-fold for GRAB-DA2m) and rapid kinetics (e.g., 10 ms rise time for dLight1.2, 40 ms for GRAB-DA2m). We provide more details regarding intrinsic properties of sensors in Section 5.

Third, DA biosensors are genetically-encoded and in that sense are ideally suited for incorporation into existing laboratory pipelines for in vivo genetic targeting. DA biosensor constructs are relatively small and thus could easily be packaged into AAVs (gene maximum size 4.4 kb), leading to stable and strong expression in multiple brain regions thanks to strong promoters (e.g., CAG, hSyn). Transgenic animal models expressing DA biosensors are also being generated. For example, transgenic flies [60,61] and zebrafish [60] carrying GRAB-DA sensors (and flies carrying the dLight1.3b sensor [61]) are available. Thus, thanks to genetic encoding through AAVs or transgenic expression, DA biosensors can in principle be chronically imaged for several months (although we recommend characterizing the system if expression must exceed 2 months, see Section 3.3). Longitudinal measurements can be advantageous when monitoring the effects of behavioral training, development, disease states or pharmacological treatments over long durations. Of note, chronic recordings are also possible with FSCV. However, one additional advantage of GPCR sensors is the ability to (in principle) track the same individual cellular ROIs over the course of an experiment when using cellular resolution imaging techniques such as miniscope imaging or 2-photon microscopy. Another major advantage is the ability to express GPCR sensors in specific cell types using cre-driver lines and flexed vectors. For instance, a flexed AAV for dLight1.1 was previously used to target DA 1 receptor (DRD1)-expressing neurons in the mPFC [98]. This may be particularly useful to compare the responses to DA between different cell types which express different DA receptor subtypes or for example between astrocytes and neurons.

Last, GPCRs represent the largest family of membrane receptors, covering most neurotransmitters and neuromodulators. This provides for many opportunities to engineer new sensors for other neuromodulators beyond the DA system (see e.g., Reference [118]) that will match as closely as possible the molecular specificity, affinity and kinetics of the endogenous receptor. Hence, GPCR biosensors for other neuromodulators such as acetylcholine [73,74], NE [71,72] and adenosine [76] have already been established, while more advanced versions of existing DA sensors [61] or preliminary sensors for serotonin (see Reference [119]), melatonin or opioids [58] are in development (for a review see: [118]). In that respect, many of the principles for sensor choice outlined in this review also apply to other neuromodulator sensors.

### 3.3. Limitations of GPCR Dopamine Biosensors

Importantly, there are several limitations that need to be acknowledged as end-users establish new sensors in the laboratory. First, there is a risk for GPCR sensors to buffer the endogenous ligand, that is, to reduce DA availability at wild-type DA receptors. This may be particularly true for high-affinity sensors capable of ligand binding in the nanomolar range (e.g., K_d_ < 100nM). Although high-affinity sensors may be ideally suited to capture DA transients of small amplitude or in brain regions with sparse innervation, the possibility for ligand buffering needs to be considered, especially after long periods of expression (e.g., several months). Ligand buffering is less of a concern in low affinity variants which have affinity in the micromolar range, which is likely to be higher than the local neuromodulator concentration. Of note, this would also not be the case for Periplasmic Binding Protein (PBP)-based sensors (another type of biosensor) such as iGluSnFR (glutamate), iGABASnFR (GABA) or iAChSnFR (acetylcholine) [120,121,122]; although a variant for DA remains to developed (see also [119,123] for a review on PBP sensors). For instance, to verify that dLight1 sensors do not buffer endogenous DA receptor function, we evaluated the effects of dLight1 expression on DA-induced cAMP signaling in cells endogenously expressing DRD1 (See next paragraph). Finding novel effective solutions to find a balance between the needed high affinity of a GPCR sensor required for imaging endogenous release in certain brain areas and the buffering/alteration of endogenous neurotransmitter signaling is an area of great interest for future sensor development studies.

Second, because they are built from membrane receptors, DA sensors may be capable of inducing GPCR downstream signaling cascades independently. There are multiple assays to investigate this, depending on the receptor type and its downstream G-protein dependent- (Gs, Gi, Gq and others) and independent- (e.g., beta-arrestin) coupling mechanisms. DRD1 is Gs-coupled and DRD2 is Gi-coupled and thus their endogenous activation increases and decreases cAMP levels, respectively. Thus, it was important to verify that the DRD1-based dLight1.1/1.2 sensors and the DRD2-based GRAB-DA sensors did not increase [58,59] and decrease [60,61] DA-dependent cAMP responses in HEK293 cells, respectively, as has been shown in References [58,59,60,61]. Additional G-protein coupling assays can include measuring cAMP levels in cells that endogenously express DA receptors as done for dLight1 and RdLight1 [58,59], monitoring cAMP in vivo in GRAB-DA-expressing animals [61] using the Pink Flamindo cAMP sensor [124] or assessing GTP-γ-S binding [60] (a proxy for G-protein downstream activity), as done for GRAB-DA. In addition, it is important to verify that DA sensors do not engage GPCR-dependent beta-arrestin downstream pathways, which would lead to sensor internalization. This was verified for dLight1/RdLight1 using a flow cytometry internalization assay and Total Internal Reflection Fluorescence (TIRF) microscopy to verify surface expression [58,59]. This was also verified for GRAB-DA sensors [60,61] using a beta-arrestin luminescent TANGO assay [125]. Of note, another advantage of PBP-based biosensors is that, as they are bacterial-derived, they do not induce downstream signaling in mammalian systems; thus, developing PBP-based DA sensors in the future is an area of great interest.

Related to this, unlike PBP-based sensors (or FSCV), GPCR-based DA biosensors are also sensitive to ligands that target the scaffold receptor on which they are built (e.g., DRD1, DRD2 or DRD4). This affects their ability to be used in combination with DA ligands; but may also in turn represent a useful tool for drug screening onto DA receptor subtypes. We expand on this topic in Section 5.6.

Moreover, it is worth noting the turnover of DA sensors has not been fully established. Hence, most of the GPCR signaling experiments were performed in short-term time-scales, minutes to days after DA-sensor genetic expression [58,59,60,61]. The absence of sensor internalization found in these studies implies that sensor expression will likely keep increasing over time which could lead to membrane overcrowding and possible toxicity or alterations in cell firing, as has been described for GCaMPs (see e.g., References [7,9,126]). Thus, if sensors need to be expressed chronically for many months, it would be advisable to reassess the absence of GPCR downstream signaling, as long-term sensor overexpression may affect these measures. A possible counter-solution to this would be to titrate the AAV titer to prevent overexpression.

It is also important to note that existing DA sensors are expressed ubiquitously throughout the membrane. One advantage of the membrane expression means that sensors sense DA transients only in close proximity to the postsynaptic cell of interest. This is different to FSCV and microdialysis which may also detect extra-synaptic DA dynamics. However, since membrane expression is ubiquitous, DA sensors do not necessarily reflect the exact location where DA receptors are located. Thus, end-users must keep in mind that dLight and GRAB-DA sensors may potentially detect levels of DA to which endogenous DA receptors may not be exposed to. Current models posit that DA is released through volume transmission, diffusing to many cells over large areas (5 to 12 μM in normal animals [127,128]) and slow timescales, well beyond the synapse (striatal DA synapses are < 0.6 μm in size; striatal intersynaptic distance: 3.5 μm [128]) (for a review see References [90,95]). Thus according to this model, DA receptors (and dLight/GRAB-DA sensors) expressed extra-synaptically at large distances from presynaptic release sites may still detect DA (albeit at diluted concentrations [128]). However, emerging evidence indicates that DA release is highly heterogeneous, with variable release dynamics spanning slow and fast time scales [18,129] and hotspots of short-lived peaks of DA confined in space [57] as well as many silent release sites [95]. A better understanding of DA receptor location and DA release spatiotemporal heterogeneity is thus warranted to fully dissect DA spatiotemporal release dynamics in the brain. New and existing tools with high spatial resolution such as FFNs [56,86] and carbon nanotubes [57,89] and possibly DA biosensors as well [58] should allow us to answer some of these important questions. Moreover, future developments in the field of DA biosensors allowing to restrict sensor expression to synaptic sites instead of ubiquitously throughout the membrane could allow to further dissect these questions.

Related to this, current DA sensors also exhibit relatively low basal fluorescence levels (brightness). They have been successfully used in 2-photon and miniscope imaging experiments in mice [58] and Drosophila [60,61], for example demonstrating heterogeneity of cortical DA release sites at high spatiotemporal resolution (17 μM ROIs) in mice. However, current sensors still lack the brightness to allow the identification of biologically relevant ROIs such as individual cells or dendritic spines. Future developments in this direction are thus warranted, following the steps of GCaMPs [8] or PBP sensors (e.g., iGluSnFr [120]).

## 4. Regional Heterogeneity of Dopamine Systems Across the Brain

In this section we will provide an overview of DA concentrations in rodent brains. We will also offer insights into what contributes to the divergence of reported values and will show why one single DA sensor is likely not suitable for application across all brain regions (see Section 5.1.3 and Section 5.1.4).

We first included classical biochemistry data of DA/NE neurotransmitter levels in various brain regions. We next performed a literature search for results derived from in vivo brain microdialysis experiments and, when available, FSCV. It is worth mentioning here that the presented data is by no means intended to present a meta-analysis of different DA concentrations but is rather intended to give a brief overview on previously published DA concentrations across the rodent brain. We note that it may be useful for end-users to seek out further published work investigating DA levels in their brain ROI and animal model of interest, for example, rodents, zebrafish or drosophila [60,61].

### 4.1. Mapping Dopamine Average Content Using Biochemistry

**Figure 2:**
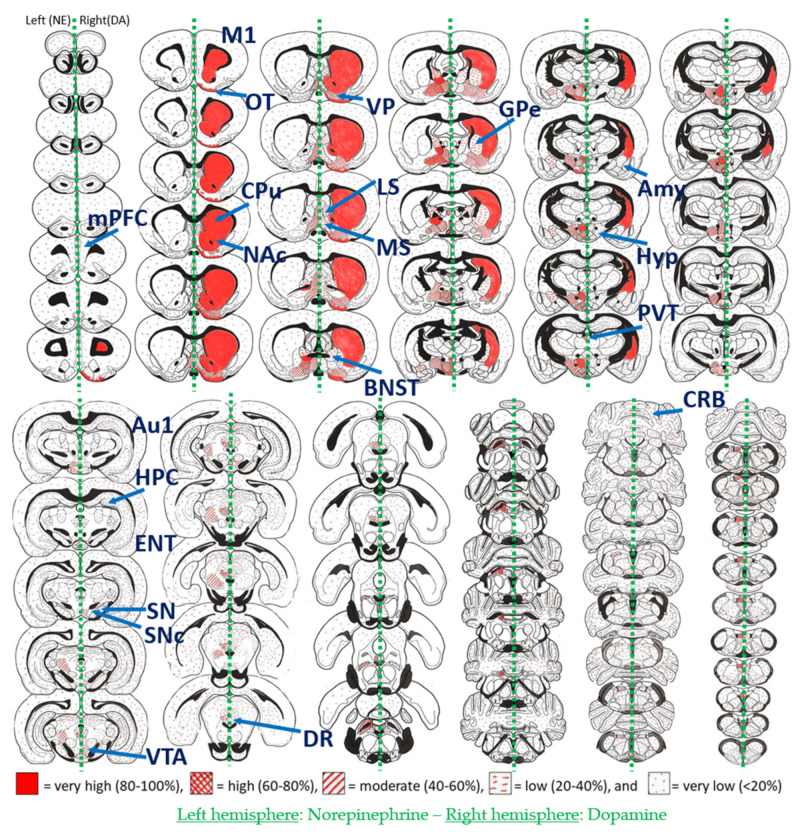
Distribution of norepinephrine (NE) (left hemispheres) and dopamine (DA) (right hemispheres) neurotransmitter levels as measured by enzyme isotope biochemistry assays in micropunches of the rat brain. The red fill pattern indicates the percentage of NE and DA relative to the highest measured value. NE: very high = greater than 64.0; high = 48.1–64.0; moderate = 32.1–48.1; low = 16.0–32.1; very low= less than 16 ng/mg protein. DA: very high = greater than 83.6; high = 62.7–83.6; moderate = 41.8–62.7, low = 20.9–41.8; very low= less than 20.9 ng/mg protein. Of note, the distribution pattern of DA and NE in the dorsal striatum and NAc is dramatically distinct—with DA content being very high (80–100%), while NE content is very low (less than 20%). Au1: primary auditory cortex; BNST: Bed nucleus of the stria terminalis; CPu: caudate-putamen (= dorsal striatum); CRB: cerebellum; DR: dorsal raphe; ENT: entorhinal cortex; GPe: globus pallidus externus; HPC: hippocampal formation; Hyp: Hypothalamus; LS: lateral septum; M1: primary motor cortex; mPFC: medial prefrontal cortex; MS: medial septum; NAc: Nucleus accumbens; OT: olfactory tubercle; PVT: paraventricular nucleus of the thalamus; SN: substantia nigra; SNc: SN compacta; TeA: temporal association cortex; VP: ventral pallidum; VTA: ventral tegmental area. For detailed brain region annotations see the original image source [130]. This Figure was modified with permission from Björklund & Hökfelt (1984), Handbook of Chemical Neuroanatomy, Vol. 2–Part 1. © Elsevier Science Publishers B.V. (1984), Amsterdam, Netherlands [130].

We first looked at the biochemical content of DA/NE neurotransmitters throughout the brain to gain insights into which areas are likely to receive innervation from DA, NE or both. We focused on classical studies performed by Björklund & Hökfelt [130] who quantified DA/NE content (ng protein/mg of tissue) using micropunches and enzymatic isotope assays. The main advantage of these biochemistry studies is the ability to gain a quick overview of the entire brain’s neurotransmitter content in a molecular specific fashion (DE can be distinguished from NE). Such biochemical maps have been indispensable for early insights into DA/NE systems and later on for guiding tool development for DA/NE measurement in vitro or in vivo.Biochemical maps are presented in Figure 2, illustrating the divergent distribution patterns of DA (right-hand side of brain section) and NE (left-hand side) in the rat brain. Since the striatum contains dense levels of DA but weak levels of NE, it is widely accepted that electrochemical techniques such as FSCV performed in this brain region report DA signaling with negligible contribution of NE signals. It can be further appreciated from Figure 2 that a multitude of other regions in the rat brain receive DAergic inputs of varying magnitude. This fact illustrates that the requirements for DA sensors may vary largely across different brain regions. It needs to be emphasized, however, that these biochemical insights do not directly allow us to draw conclusions on the cellular compartment of DA/NE (e.g., cell body, axon, terminal) or release dynamics of such neurotransmitters. We refer to neuroanatomical mapping studies that provide useful complementary information regarding DA and/or NE cellular/axonal/terminal expression patterns throughout the brain [45,131,132]. More insights on in vivo release dynamics are provided in the next sections.

### 4.2. Mapping Basal Dopamine Levels Using In Vivo Microdialysis

**Figure 3:**
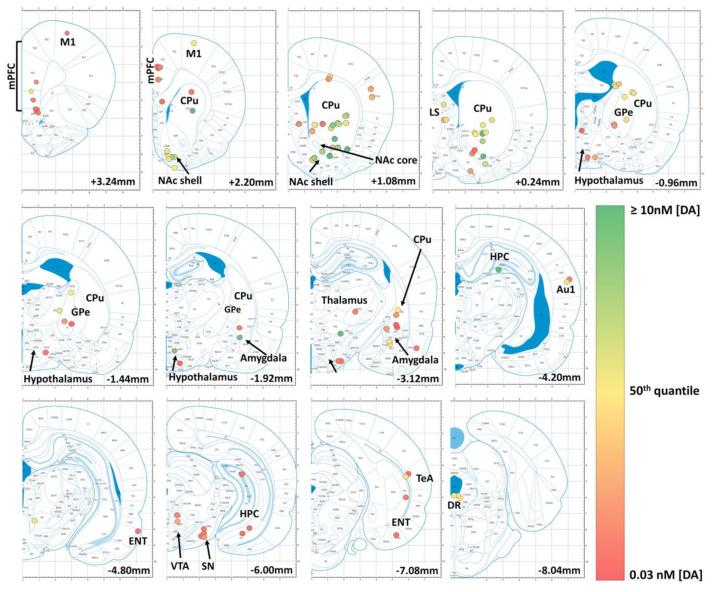
Graphical representation of previously reported basal dopamine concentrations measured via intracerebral microdialysis experiments performed in rats. Coronal brain sections from AP + 3.24 mm to AP -8.04 mm (relative to bregma) are depicted. The colored circles indicate the reported positions of the microdialysis probes and the fill color of the circles is color-coded to represent the values of the reported basal dopamine concentrations. Of note, the striatal basal dopamine concentrations are amongst the largest in rat brain (brain sections AP + 1.08 mm and AP + 0.24 mm). Importantly, the basal dopamine levels do not represent phasic dopamine release since they are predominantly measured during baseline at rest and are a result of dialysate collection times in the range of several minutes. For detailed information on the previous studies reported in this analysis please consult Supplementary Table S1. This figure was modified with permission from Paxinos & Watson (2005), The rat brain in stereotaxic coordinates, 5th edition. © Elsevier Academic Press (2005), Burlington, MA 01803, USA [213].

We next focused on in vivo microdialysis data obtained from a selection of rodent studies (Figure 3). The major advantages of microdialysis are: (i) its nanomolar sensitivity and (ii) its molecular specificity, allowing to distinguish DA from NE in microdialysis samples using HPLC. For more details on microdialysis techniques see Section 2.1. In Supplementary Table S1 we list basal DA concentration from different microdialysis studies across various brain regions including the reported parameters used [42,48,81,133,134,135,136,137,138,139,140,141,142,143,144,145,146,147,148,149,150,151,152,153,154,155,156,157,158,159,160,161,162,163,164,165,166,167,168,169,170,171,172,173,174,175,176,177,178,179,180,181,182,183,184,185,186,187,188,189,190,191,192,193,194,195,196,197,198,199,200,201,202,203,204,205,206,207,208,209,210,211]. Since microdialysis-derived values can vary greatly, we calculated the median values of all the reported basal DA concentrations for each brain region and normalized it to the median value derived from the dorsal striatum (3.75 nM), presented in Figure 3. For details on the actual reported values per study and the parameters used, see Supplementary Table S1. Of note, the predicted basal DA level from asynchronous tonic firing has been reported around 10–30 nM [11,212], in the range of basal DA levels measured by microdialysis in the striatum/NAc (Figure 3).

Based on this analysis, we found that basal DA levels in the NAc reached ~62% relative to the dorsal striatum, followed by DA levels in the lateral septal nuclei (~51%) and the dorsal raphe nuclei (40%). The pallidum and ventral tegmental area (VTA) reached ~21% and the amygdala ~15% relative to the dorsal striatum. Furthermore, median values in the hypothalamus, thalamus, substantia nigra and cerebral cortices all were at ~7% of the median dorsal striatal values. The lowest median DA concentrations reported in our selection were in the hippocampus with ~3% relative to the dorsal striatum. In most of these referenced studies, basal DA concentrations were in the single-digit nM range. This illustrates that basal DA levels can reach 10–30 fold lower values as compared to the striatum in certain brain regions, further supporting the notion that different sensors will be best suited for individual brain regions according to sensor affinity.

It is worth noting, however, that microdialysis is limited in its ability to detect phasic DA release events. Our microdialysis maps (Figure 3) must therefore be interpreted with caution. Indeed, from our personal experiences and communication with other dLight1 users, we know that dLight1.1 and dLight1.3b perform well in the striatum, all the while having DA affinities (dissociation constants Kd) of 330 nM and 1.6 μM, respectively [58]. Hence basal DA levels reported by microdialysis (single-digit nM range) largely underestimate phasic DA release events (see Section 4.3). 

This is due to the inherently slow time resolution of microdialysis due to long sample collection times. Considering that phasic DA release events are in the sub-second scale and are therefore only elicited in a subsection of the total microdialysis recording time (minutes), it is evident that averaging DA concentrations in microdialysis samples over several minutes will drastically diminish reported values and can therefore not represent the actual peak amplitudes of DA transients. Moreover, reported microdialysate concentrations depend on other parameters, including the extraction efficiency (i.e., relative recovery), which is influenced by the perfusion flow rate, dialysis membrane properties (e.g., active membrane length), probe geometry and physiological processes (transport, uptake, release, binding, metabolism) [214]. As these parameters must be optimized for the purpose of each individual experiment, there is a large heterogeneity in reported parameters and hence also in the reported DA concentrations, which hinders comparison between studies. For better transparency and comparability, we have reported most of these parameters in Supplementary Table S1.

### 4.3. Insights into Phasic Dopamine Levels Using FSCV

In a last step, we looked at FSCV studies measuring catecholamine levels in behaving rodents. The major advantages of FSCV are: (i) it allows us to track changes in metabolite levels with a much higher temporal resolution (subsecond) as compared to microdialysis, (ii) its nanomolar sensitivity and (iii) its quantitative nature (unlike DA biosensors [118]). Since FSCV captures fast catecholamine transients, the reported peak concentrations are usually much larger vs. microdialysis studies. One limitation of FSCV is that it does not distinguish DA from the closely related NE (see Section 2.1 for more details), so that reported levels report a mix of DA/NE release in regions with dual innervation. For an elegant strategy to counter this, see below and [23]. Note that the majority of studies using FSCV were performed in the NAc or striatum, with little work performed outside these brain regions due to the lack of molecular specificity. Thus, we did not have sufficient data to provide a reliable anatomical map. Werely on a few examples below to gain insights into the peak DA concentration range (nM) found in these regions under native and stimulated conditions. 

For instance, native DA release concentrations were reported by Robinson et al., (2002): they found peak concentrations of 212 nM and 290 nM in the dorsal striatum and NAc, respectively, during social interactions of male and female rats [215]. Since this data was recorded in the striatum (with negligible NE innervation), the reported catecholamine levels can be considered as peak DA levels. Moreover, Hamid et al., (2016) reported increases in DA around 20 to 50 nM in the rat NAc as rats approached rewards or upon reward cues [216]. Roitman et al., (2004) reported 50–100 nM DA release upon exposure to learned cues or lever presses [217]. Philips et al., (2003) found NAc DA increases around 50–150 nM during cocaine seeking behavior in rats [218]. Interestingly, Howe et al. (2013) found comparable levels (5–40 nM) of DA released in a prolonged manner (5–10 seconds ramps) as rats approached distant rewards [219]. There are only a few FSCV studies outside the striatum/NAc. Using an elegant approach Vander Weele et al. (2018) showed behaviorally-induced increases in catecholamines in the mPFC that can be attributed to DA. Indeed, authors found that a tail pinch led to a 20–40 nM increase in catecholamines in the mPFC, an effect that was largely blunted following photoinhibition of VTA DA neurons [23]. 

Of note, evoked DA release can also be measured upon stimulation of DAergic neuronal populations by either electrical or optogenetic stimulation to evaluate maximal DA release capacity. Peak DA concentrations elicited by these two modalities reach higher levels vs. native release, depending on the stimulation parameters applied. For example, Wightman et al., (2007) found DA concentrations reaching up to 1500 nM in rats upon electrical VTA stimulation (2 ms, 24 pulses, 60 Hz, 125 µA) [220].Oleson et al., (2009) reported similar values obtained from mice after electrical VTA/substantia nigra pars compacta (SNc) stimulation (2 ms, 24 pulses, 60 Hz, 120 µA) reaching ~1000 nM peak DA transients before single cocaine injection and ~2000nM after a single cocaine injection [221]. Philips et al. (2003) also found increases in DA around 600–700 nM in the NAc following a similar electrical stimulation protocol (VTA, 24 pulses, 60 Hz, 120 µA) [218].

Using brief VTA optogenetic stimulation (10-ms pulses, 60 pulses, 30 Hz, 0.5 s epoch), Hamid et al., (2016) reported increases in DA around 120 nM in the rat NAc [216]. Bass et al. (2010) reported a power-, pulse- and frequency-dependent release in the rat striatum following SNc optogenetic stimulation. For instance they found release around 45 nM for weak stimulation protocols (e.g., 4-ms pulses, 20 pulses, 20 Hz, 1 sec epoch) and up to 500 nM for strong stimulation protocols (20-ms pulses, 60 pulses, 60 Hz, 0.5 to 1 sec epoch) [222]. Moreover, VTA optogenetic stimulation (60 pulses, 20 Hz) also led to DA release around 40 nM in the mPFC [23]. Interestingly, optogenetic stimulation of DA neurons in Drosophila using continuous (3 to 7 s) blue light led to comparable DA levels (600–800 nM, measured by FSCV [223]) as in the rat. Whether behaviorally-induced release of DA in these two species is also similar or whether they differ remains to be investigated. 

## 5. Practical Considerations for Sensor Choice: One Sensor Does Not Fit All

For end users, choosing the most appropriate sensor for a given experimental application is paramount. In Section 4, we mapped DA levels across multiple brain regions in rodents as a guide for future users. Local DA levels can be a useful measure to optimize sensor choice based on ligand affinity. Dynamic range and molecular specificity are other key parameters to consider. We outline below how these sensor properties are measured and how they can guide sensor choice. Moreover, behavioral constraints, imaging acquisition methods (fiber photometry, 1 and 2-photon micro/endoscopy, etc.), multiplex experiments and pharmacological paradigms may further affect sensor choice based on other sensor properties including: kinetics, brightness, spectrum and molecular scaffold, which we further discuss below.

### 5.1. Matching Brain Region Dopamine Levels to Sensor Affinity and Dynamic Range

#### 5.1.1. Affinity

**Figure 4:**
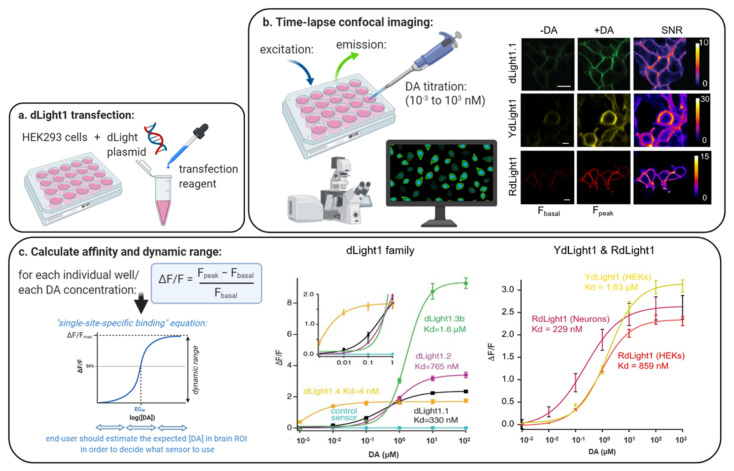
Concentration-response curves to determine affinity (K_d_) and dynamic range (dFF_max_) of dopamine (DA) biosensors in vitro. (**a**) In a first step, HEK293 cells are transfected with the DA sensor plasmid of interest. (**b**) After 2 days of sensor expression, sensor fluorescence is measured in response to DA titrations (10^−3^ to 10^3^ nM) which are perfused into the bath during time-lapse confocal imaging with 488 nm (dLight1, YdLight1) or 561 nm (RdLight1) light. Time-lapse images of fluorescent cells before and after addition of increasing DA concentrations are obtained and used to calculate the signal to noise ratio (SNR) and average fluorescence intensity (F). (**c**) dFF is calculated using the following equation: [F(peak)–F(basal)]/F(basal) where F(basal) and F(peak) are the averaged fluorescence intensity of 10 frames before and after, addition of a given DA concentration, respectively. Maximal dFF values for a given concentration are plotted and a single-site-specific binding equation is used to fit the data points and determine the affinity (K_d_ value) and dFF_max_ of the sensor for this ligand (titration data shown in panel c are from References [58,59]). For further details on the protocol, see Reference [99].

The sensitivity of a GPCR sensor for its neuromodulator, such as DA, primarily depends on two main properties: its affinity and dynamic range. The ligand affinity of a sensor corresponds to the neuromodulator concentration required to reach half the maximal fluorescent response (also referred to as dynamic range, dFFmax) and is expressed as an apparent dissociation constant (K_d_) or half-maximal effective concentration (EC_50_). Figure 4 outlines how affinity and dynamic range can be calculated. Ligand affinity gives an indication of the physiological concentration of DA at which the sensor will be most sensitive and can thus help in the selection of sensors for in vitro or in vivo applications based on regional DA concentrations (see Section 4 for our in vivo estimates in rodents). 

**Figure 5:**
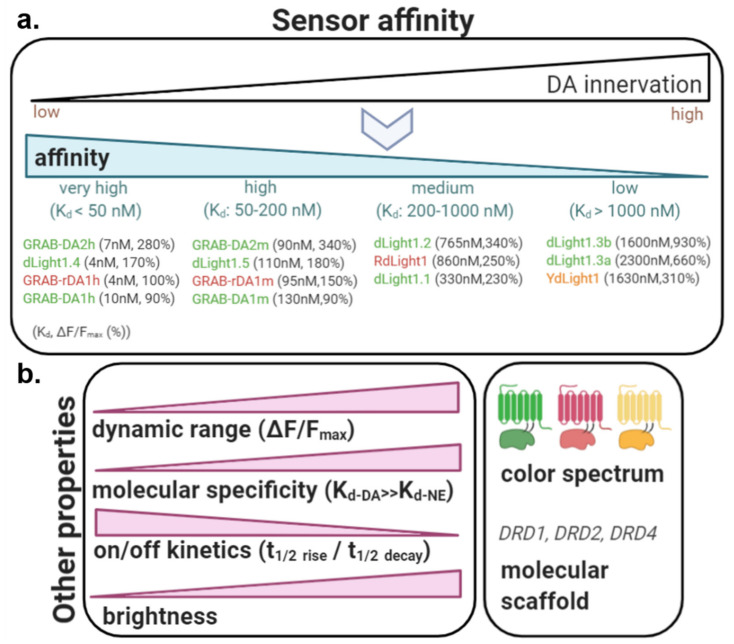
Sensor properties to consider for sensor choice: (**a**) Local dopamine (DA) levels can be a useful measure to guide sensor choice based on ligand affinity. It is generally recommended to look at sensor affinity as a first criterion and to match it with the local expected concentration of DA in the brain ROI, using high and very high affinity variants for regions with sparse innervation and medium/low affinity variants for regions with dense innervation; here we classify existing DA sensors based on their affinity category (affinity K_d_ and dynamic range dFF_max_ are noted in brackets). (**b**) Other sensor properties should be considered (see also Table 1 for exact values) and their relative importance will depend on the individual experimental parameters: (i) dynamic range (if possible at least 250–300%), (ii) molecular specificity (affinity for DA should be far greater than affinity for NE in brain regions with dual DA/NE innervation), (iii) on and off kinetics (should be as short as possible in assays where high temporal resolution is required), (iv) basal brightness (may not matter for fiber photometry but high brightness may improve identification of small cell compartments in 1- and 2-photon imaging), (v) color spectrum (3-colors available; important when multiplexing with opsins, sensors or photopharmacology) and (vi) molecular scaffold (3-scaffolds available; important when multiplexing with (photo)pharmacology: if drug has affinity for the DA receptor scaffold, signal may be affected; this property can however be harnessed in drug discovery experiments where sensor fluorescence can be used as a specific readout of DA receptor subtypes activation in vitro or in vivo).

In an ideal scenario, multiple sensor variants will exist covering a broad range of affinities and dynamic ranges to match the demands of each individual system or brain region being studied. Currently, sensors have been successfully engineered against DRD1 (dLight1.1, 1.2 and 1.3, YdLight1, RdLight1), DRD2 (dLight1.5 and all GRAB-DA sensors) and DRD4 (dLight1.4) as the GPCR molecular scaffold, but not yet for the DRD3 and DRD5 subtypes due to expression issues [58]. The molecular scaffold directly affects the sensor affinity and dynamic range. One main advantage of using native GPCRs as the molecular backbone, is that they leverage the evolutionary specificity for the ligand of choice and thus naturally lead to affinities well within the physiological range (as opposed to bacterial PBP-based sensors [123]). As shown in Table 1, the dLight1/RdLight1 family covers affinities from very high, single-digit nanomolar affinities (e.g., dLight1.4: 4nM), high (dLight1.5), medium (e.g., dLight1.1, 1.2; RdLight1) and low (dLight1.3a, 3b; YdLight1) affinities [58,59]. GRAB-DA sensors also include very high (DA1h, DA2h, rDA1h) and high (DA2m, rDA1m, DA1m) affinity variants [60,61] (Figure 5). 

#### 5.1.2. Dynamic Range

The dynamic range (dFF_max_) is defined as the ratio between the estimated maximal fluorescent response upon ligand addition and the baseline fluorescent level (before ligand addition) (Figure 4). It thus provides an estimate of the range of responses that can be obtained against varying DA concentrations. A large dynamic range critically determines a sensor’s sensitivity to small and large fluctuations in DA levels. In our original publications, we found dLight1/RdLight1 variants to exhibit close to 200% and up to 900% dynamic range [58,59], matching levels found for PBP-based sensors [119]. Although the first generation of GRAB-DA sensors (DA1h, DA1m, rDA1h, rDA1m) exhibited a 2 to 10-fold lower dynamic range vs dLight sensors, the second generation provides enhanced levels (340% for DA2h, 280% for DA2m) [60,61] (Figure 5).

Affinity and dynamic range are typically assessed in HEK293 cells expressing the GPCR sensor by measuring fluorescent responses (dFF) in response to bath application of increasing (pM to μM) concentrations of DA followed by data fitting against a single-site specific binding equation or a Hill Equation (see e.g., Reference [58]) (Figure 4).

#### 5.1.3. Sensor Choice Based on Affinity and Dynamic Range

For future sensor users, it is generally recommended to look at sensor affinity as a first criterion and to match it with the local expected concentration of DA in the brain ROI (Figure 5). This is especially true in experiments looking at behavioral-induced DA release. This may be less essential in experiments solely focusing on electrically- or optogenetically-evoked DA release, where detected DA levels are often found to be several fold higher as compared to endogenous release (see Section 4.3). Next, among the sensors selected, it is best to favor a sensor with maximal dynamic range (if possible, >250–300%). We found that dLight1.1, dLight1.2 and RdLight1 displayed an optimal combination of medium ligand affinity (330 nM, 765 nM and 860 nM, respectively) and good dynamic range (230%, 340% and 250%, respectively), making them ideally suited for in vivo bulk (photometry) imaging of large DA transients in response to rewards or cues in the heavily innervated striatum or NAc [58,59], see also: [107,110,111,112,113,115,224,225]. On the other hand, sensors with high and extremely high affinity such as GRAB-DA2m (K_d_= 90 nM; dFFmax = 340%; validated in vivo), GRAB-DA2h (K_d_ = 7 nM; dFFmax = 280%; validated in vivo) [61] and dLight1.4 (K_d_ = 4.1 nM; dFFmax = 170%; not validated in vivo yet) [58]; as well as other sensors in development (not shown) are ideally suited to detect DA release in brain regions with sparser and extremely sparse innervation or may potentially be used to track tonic DA changes in the nanomolar range [11] (this remains to be determined). Their in vivo applications in such brain regions remains to be demonstrated, however, along with their ability to distinguish DA from NE when dual innervation patterns arise (see next section on Molecular specificity). These sensors may also work in densely innervated regions, albeit some possible saturation upon strong DA release events. Finally, dLight1.3a and dLight1.3b variants display a combination of low affinity (K_d_ > 1000 nM) but very higher dynamic ranges (2–3 × higher than that of dLight1.2). dLight1.3b has already demonstrated its use in vivo in the NAc using fiber photometry [101,108,226]. Because of the higher threshold of DA concentration required for sensor activation, these sensors are less well suited for brain regions with low DA. However, by remaining insensitive to small DA variations (e.g., tonic DA variations or variations arising in more sparsely innervated sites) but responding with large signals where DA is heavily released (e.g., in response to highly salient behavioral stimuli or in subregions heavily innervated) these variants may be ideally suited to detect and visualize DA release hotspots using miniscope, 2photon or even mesoscopic techniques, although these applications remain the be tested for this purpose. Moreover, combining medium affinity variants (e.g., RdLight1) with low affinity variants (e.g., dLight1.3b) may allow other people to gain a deeper understanding of the spatiotemporal dynamics of DA release, diffusion and uptake.

Note there is the distinct possibility for high-affinity sensors (nanomolar range) to bind DA and induce ligand buffering, especially after long periods of expression (e.g., several months). Ligand buffering is less of a concern in low affinity variants (micromolar range) with affinity values higher than the local neuromodulator concentrations. Developing novel sensors with the perfect balance of high affinity and low risk for ligand buffering is an area of great interest for future studies. 

#### 5.1.4. Published In vivo Validations of DA Sensors in Brain Regions with Dense vs. Sparse Innervation 

In Supplementary Table S2 we present an overview of previously published in vivo applications of DA biosensors from the dLight, RdLight and GRAB-DA families in rodent animal models [38,58,60,61,98,101,102,103,104,105,106,107,108,109,110,111,112,113,114,115,116,224,226,227,228]. The large majority of experiments was performed in either the dorsal striatum or the NAc where the DA concentration reaches its maximal levels. Studies have successfully used high, medium and low affinity DA variants in these regions (see Figure 5), although the medium variants (e.g., dLight1.1/1.2) are the most commonly used. High-affinity variants could help detect subthreshold DA release dynamics but are likely to saturate while low-affinity variants could be useful to detect hotspots but will omit smaller DA transients. Based on our brain maps, we predict that medium affinity variants are likely to work well in other brain regions with high DA content, that is, the olfactory tubercle.

DA biosensors have also successfully been put to use in brain regions with medium to low DA, albeit with reduced signal to noise ratio. This includes the frontal/motor cortex (dLight1.1 & 1.2) [58], the mPFC (dLight1.1) [98], the basal (dLight1.1 & 1.2) and central (GRAB-DA2m) amygdala [38,98,103] and the bed nucleus of the stria terminalis (GRAB-DA2m) [38]. These studies used sensors with medium to high affinities (90–765 nM) for DA and maximal dFF between 230–340%. 

In future work, recently developed sensors with very high affinities (GRAB-DA2h, -rDA1h and dLight1.4) could improve signal detection in these brain regions. To the best of our knowledge, very high-affinity sensors have thus far not been applied outside of the striatum in vivo. This might be due to the lower dFF_max_ values reached by these sensors. The candidate with the largest dFF_max_ value (280%) within the group of very high affinity DA-sensors is GRAB-DA2h and might at this point be the most likely candidate to be successfully applied in vivo in brain regions with low DA innervation. One exception are brain regions with dual DA/NE innervation like the mPFC where dLight variants are preferred (see Section 5.2). 

Upon comparison with our anatomical DA concentration maps (see Figure 2 and Figure 3 and Supplementary Table S1), we propose that existing high and very high affinity DA biosensors are likely to perform well in brain areas with moderate or low DA levels (e.g., ventral pallidum, dorsal raphe, amygdala, medial and lateral septum, hippocampus, cortex or GPe). This needs to be tested empirically, however, given the limitations of our biochemistry/microdialysis maps (See above). Medium affinity variants may also work in these regions but likely with a weaker signal (lower dFF). Future work is warranted to further boost the dynamic range of high and very-high affinity DA-sensors for these applications.

### 5.2. Sensor Molecular Specificity Matters in Brain Regions with Dual Dopamine/Norepinephrine Innervation

Another property to take into consideration includes the sensors’ ligand specificity for DA over NE (and other monoamines), as the chemical structure of DA vs. NE is very similar. For instance, DA and NE only differ by one hydroxyl group [229] and many currently available methods to record DA release such as FSCV, FFNs or carbon nanotubes cannot differentiate DA from NE. To determine sensor molecular specificity, DA sensor affinity for other monoamines such as NE can be titrated in HEK293 cells using the same methods as described in the previous section. Doing this, the affinity for DA over NE was found to be 70-fold higher for dLight1.1 [58], 60-fold higher for RdLight1 [59] and 270-fold higher for dLight1.3 b [101]. Further, dLight1.1 affinity for DA over epinephrine was 40-fold higher and responses to other neuromodulators were negligible. Thus, one of the main advantages of dLight1 GPCR sensors over other available methods is the ability to reliably distinguish DA from NE. This is particularly relevant in brain regions that harbor dual innervation of both DA and NE systems, such as the mPFC [23] or the PVT [39], among others. Notably, molecular specificity of all GRAB-DA sensors for DA over NE was 10 to 20-fold. In the striatum and NAc, where NE innervation is negligible [230], first and second generation GRAB-DA sensors are likely to be highly specific to DA. However, when considering experiments in other brain regions, careful estimations of NE basal and evoked levels must be made. For instance, in the mPFC, basal levels of NE are in the single-digit nM range but evoked NE levels can be found in the several hundreds of nM, often found to be higher than DA levels, depending on the context [23,231,232,233,234]. Thus it is important to keep in mind that the high-affinity variants DA1h, DA2h and rDA1h have a ligand affinity for NE around 100 nM, which is likely to detect NE in the mPFC, although this remains to be tested (Of note: medium-affinity variants DA1m, DA2m and rDA1m sensors have a ligand affinity for NE around 1 to 2 μM). In contrast, the medium-affinity dLight1.1 variant had an affinity for NE at 19,850 nM [58]. When in doubt, DA sensors responses can be titrated ex vivo and in vivo in response to noradrenergic drugs and/or locus coeruleus optogenetic stimulation to ascertain molecular specificity.

### 5.3. Maximizing Sensor Kinetics Allows to Sparse out Individual Transients in Response to Closely Related Stimuli

The rise (t_1/2_ rise) and decay (t_1/2_ decay) times represent the times required by the sensor to (1) reach half of the peak activity following acute stimulation and (2) return to half of the basal level prior to simulation. The in vitro on- and off-kinetics of the first generation of dLight1 sensors are excellent (e.g., dLight1.1: t_1/2_ rise: 10 ms and t_1/2_ decay: 100 ms; RdLight1: t_1/2_ rise: 14 ms and t_1/2_ decay: 400 ms) surpassing existing GCaMP sensors (e.g., GCaMP7f: t_1/2_ rise: 27 ms and t_1/2_ decay: 265 ms). Moreover, dLight1.1 and RdLight1 kinetics were also evaluated in vivo, demonstrating highly accurate reporting of individual stimulation events in the NAc at 5 Hz (dLight1.1) and 2 Hz (RdLight1) and good reporting at 10 Hz (dLight1) and 4Hz (RdLight1) following VTA optogenetic stimulation (2 s total) [58,59] (see also Figure 1). In vitro on-kinetics of GRAB-DA sensors are in the same order of magnitude as dLight1.1/1.2 (e.g., GRAB-DA2m: t_1/2_ rise: 40 ms) but off-kinetics are 7 to 70 times higher as compared to dLight1 (e.g., GRAB-DA1m: t_1/2_ decay: 710 ms), making them less attractive to detect rapidly succeeding DA transients. We must acknowledge, however, that on and off kinetics are measured using different methods across laboratories, making it difficult to make precise comparisons. The on- and off-kinetics of dLight sensors were measured following electrical stimulation in brain tissue [58,59] where endogenous reuptake mechanisms can rapidly decrease DA concentrations and in turn reduce off-kinetics. This contrasts with GRAB-DA sensors where the same properties were measured following a DA puff in HEK293 cells that lack these rapid reuptake mechanisms [60,61]. 

On- and off-kinetics are critical parameters to consider when choosing sensors. Using sensors with short on- and off-kinetics is most important in assays where high temporal resolution is needed and acute DA transients are expected (alternatively, one could consider changing the behavioral parameters to fit the kinetics of the sensor of interest). For instance, behavioral tasks assessing reward behavior encompass cues followed by reward delivery which often occur within a few seconds from each other. Based on the in vivo frequency-dependency experiments outlined above, we expect dLight1.1 and RdLight1 to track events with intertrial intervals of up to 0.5 and 0.2 seconds, respectively; at least under the experimental conditions used in References [58,59]. On the other hand, slower sensor kinetics may presumably allow to detect smaller DA transients via summation and signal integration; this needs to be accounted for during data interpretation but may improve sensitivity (although this remains to be tested empirically). This would be akin to GCaMP6s (or GCaMP7s) which demonstrated slower kinetics vs. GCaMP6f (or GCaMP7f) but higher sensitivity (= higher response to 1 or 10 AP trains) and is partly due to their 2-3 fold higher affinity for calcium [7,8]. Moreover, DA transients can also encode slower variables such as spontaneous movements across several seconds [58,94]. DA transients can also ramp up over several seconds in freely moving animals approaching rewards or running to distant goals following cues [94,101,105,216]. Here fast on/off-kinetics are useful to identify more precisely the boundaries of the transient; however slow off-kinetics may allow to detect smaller fluctuations in DA, that could otherwise be occluded. In such complex cases the best sensor remains to be determined empirically. For an interesting perspective on sensor kinetics, see Reference [235]. Of note, in assays where fast off-kinetics are less of a concern (e.g., to track slow changes over minutes/hours or measure changes in tonic levels), other sensor properties can be prioritized instead of kinetics. For example, a recent study found that injection of heroin to naïve animals induced an increase in DA release in the NAc within minutes following injection; in this experiment, fast kinetics were not required [110].

Moreover, in experimental paradigms aiming at addressing the dual dynamics of two neurotransmitters simultaneously and their possible causal relationships (e.g., DA and NE in the mPFC or DA and acetylcholine in the NAc), it would be important to choose two sensors with similar kinetic profiles (see Reference [119] for the most recent summary table of neuromodulator sensor kinetics). Currently, many of the neuromodulator sensors differ by up to a factor of 70 in their on- and off-kinetics (see comment above regarding caveats in comparing sensor kinetics). This makes it difficult to interpret possible bidirectional relationships. One option is to measure the kinetics of the chosen sensors in slice or in vivo following optogenetic or electrical stimulation to determine the lag of one sensor against the other and in turn apply corrections in subsequent analyses. When dual sensor kinetics are too different from each other, another solution is to use axon-GCaMP6f as a common denominator. For instance, one could determine relative dynamics of axonal activity of DA vs. NE afferents into the mPFC using axon-GCaMP6f; before using DA vs. NE biosensors to measure relative release dynamics. For example, we found high fidelity between axonal calcium levels arising from the midbrain (measured with axon-GCaMP6f) and DA release (measured with RdLight1) in the NAc in a reward task [59].

### 5.4. Future Developments in Sensor Brightness/Subcellular Expression will Improve 1 and 2-Photon Imaging 

DA biosensors have the potential to be deployed across multiple in vivo imaging modalities such as multiplex fiber photometry, 1-photon miniscope imaging and 2-photon regular or mesoscopic imaging and recordings can be performed repeatedly across multiple days (for details on these techniques, see References [62,68,69]). DA sensors work optimally for rodent fiber photometry and hence the majority of published work thus far was performed using this method. Indeed, the affinity, dynamic range and basal brightness levels of the first generation of DA sensors were found highly appropriate for fiber photometry applications, as demonstrated across multiple experiments and publications [38,58,60,61,98,101,102,103,104,105,106,108,109,110,111,112,113,114,115]. For fiber photometry, low basal fluorescence and high evoked fluorescence is preferred to optimize response detection. 

Notably, dLight1 has successfully been used to identify DA transients across the striatum and cortex using 2-photon imaging (Figure 1) [58].GRAB-DA1 and 2 sensors have also been used in drosophila to perform 2-photon imaging [60,61]. 2-photon imaging of dLight1.1 transients (Figure 1) demonstrated heterogeneity of cortical DA release sites as defined across 17 μM ROIs in mice. Thus, sensors were bright enough to identify behaviorally relevant fluorescent responses. Although dLight1.3b has not been validated in 2-photon imaging, we speculate that, due to its low affinity (1600 nM) and very large dynamic range (930%), it may be well-suited to detect and visualize DA release hotspots in densely innervated regions using these imaging techniques. However, it must be noted that, in published 2-photon experiments [58], it was not possible to define biologically-relevant ROIs such as individual cells or dendritic spines. Moreover, 2-photon transients were pooled across the field of view [58,60,61]. One possible solution to this caveat would be to increase both the basal and evoked brightness of existing sensors, as well as the surface expression, thus allowing to improve the detection of cellular compartments. Such tool engineering approaches have recently been implemented for calcium sensors, with the development of the bright calcium sensor GCaMP7b, engineered to provide increased resting and evoked fluorescence to allow improved imaging of small processes such as boutons [8]. Similar developments to enhance the brightness of DA sensors are ongoing, as shown in a recent preprint for the GRAB-DA2m sensor [61], which, however, remains to be validated in 2-photon rodent imaging assays. Another solution would consist in targeting the sensor to specific subcellular compartments (e.g., dendrites), as has been done recently by Broussard et al., where GCaMP6f was fused to the axon motif GAP43 to drive axonal localization and improve 2-photon presynaptic imaging [52].

### 5.5. Sensor Color Spectra Permits Dual Imaging and Multiplexing with Optogenetics and Photopharmacology

Another important factor to consider is the sensor color spectra. Given that the green DA sensors dLight1 and GRAB-DA1 and 2 were engineered using cpGFP, that is, the same fluorescent monomeric protein as GCaMPs, it is not surprising that the spectral properties are highly similar [7,8]. Using one-photon emission spectra in HEK293 cells (dLight1, GRAB-DA2) and awake mice (GRAB-DA1), authors identified peak emission around 517 nm for dLight1, 510 nm for GRAB-DA1 and 520 nm for GRAB-DA2 sensors following excitation at 488 nm (dLight1) and 470 nm (GRAB-DA1). Moreover, two-photon emission spectra for dLight1 peaked at 920 nm. The isosbestic point (=excitation wavelength at which sensor absorbance does not change in response to DA) for GRAB-DA2h is 440 nm [61] as determined by one-photon excitation spectra. Isosbestic points were not formally generated for dLight1 and GRAB-DA1; however, several laboratories determined empirically that dLight1 is insensitive to 405 nm excitation light in vivo (see e.g., raw traces in Supplement 1 Figure 1 in Reference [115]). For more details on isosbestic channels and their limitations, see Reference [62]. Similarly, red-shifted sensors RdLight1 and GRAB-rDA1 yielded emission spectra with minimal overlap with green sensors and highly comparable to red-shifted calcium sensors like jRGRECO1a (all using cpmApple as the chromophore). Peak emission is at 588 nm (RdLight1) and 595 nm (GRAB-rDAs) following 561 nm (RdLight1) and 565 nm (GRAB-rDAs) excitation (isosbestic points remain to be determined in future work).

Sensor color spectra is important to consider when multiplexing DA imaging with other methods such as optogenetics, calcium/neurotransmitter imaging or photopharmacology. Previous work by us and others found that the green sensors dLight1.1 (Figure 1), dLight1.3b, GRAB-DA1h, 1m and DA2h, 2m are compatible with red-shifted optogenetic stimulation using fiber photometry in rodents. Hence, robust increases in fluorescence in the NAc or dorsal striatum were detected in response to VTA or SNc red-shifted (ChrimsonR, C1V1) optogenetic stimulation [58,60,61,226]. Moreover, we found that RdLight1 is compatible with optogenetic stimulation with the green opsin ChR2 (Figure 1) [59]. Green and red sensors were also compatible with two color imaging, due to their orthogonal spectra. For instance, green dLight1.1 was successfully imaged in combination with the red-shifted calcium indicator jRGECO1a [77] (Figure 1) [58]. RdLight1 was imaged in the NAc in combination with multiple green sensors including axon-GCaMP6f [52] to image arising VTA DA terminals or mPFC terminals, GCaMP6f [7] to image DRD1-expressing neurons in the striatum and iGluSnFR [120] to image incoming glutamatergic inputs [59]. As mentioned previously (Section 5.3), when imaging two sensors simultaneously, sensor kinetics should be matched as closely as possible to optimize data interpretation; for instance we found that axon-GCaMP6f imaging of VTA axons and RdLight1 imaging of DA release were highly correlated during a reward task [59], which is consistent with their relatively similar kinetics (RdLight1: t_1/2_ rise: 14 ms and t_1/2_ decay: 400 ms; untargeted-GCaMP6f: t_1/2_ rise: 45 ms and t_1/2_ decay: 140 ms [7]). We do expect that VTA axonal activity (axon-GCaMP6f) and DA release (RdLight1) may be decorrelated under certain conditions, for instance when activating nicotinic acetylcholine receptors (nAchR), which are known to modulate DA release partly independently of DA axon activity (see e.g., Reference [11]). 

Moreover, in principle, green and red sensors should also be compatible with recently developed photopharmacological tools, such as Opto-XRs, that permit region or cell-specific pharmacological manipulations of desired receptors or ion channels in combination with light (for a recent review, see: [236]), although their combined applications in vivo remains to be tested. For instance, blue light illumination of a chimeric rhodopsin-DRD1 receptor (Opto-D1R) expressed in NAc DRD1-expressing cells increased DRD1-expressing neuron activity and promoted social interaction in mice [63].

It should also be noted that red-shifted DA variants may be particularly useful in applications where afferent calcium activity needs to be imaged simultaneously. Indeed, although red-shifted calcium sensors are highly effective for imaging local somatic activity, their heightened susceptibility to photobleaching makes them less than an ideal choice for imaging axonal activity. For such situations, green calcium sensors such as axon-GCaMP6f [52] or GCaMP7 [8] are preferred, in combination with red-shifted DA sensors. Moreover, due to their increased light penetration and imaging depth, red-shifted sensors may produce enhanced DA signal quality, which we observed in fiber photometry recordings (see: [59]). This could be an added advantage in applications such as through-cranium imaging applications or to increase maximal imaging depth [237], although this remains to be demonstrated in vivo.

### 5.6. Molecular Scaffold as a Double-Edged Sword for Pharmacology and Drug Discovery

**Figure 6:**
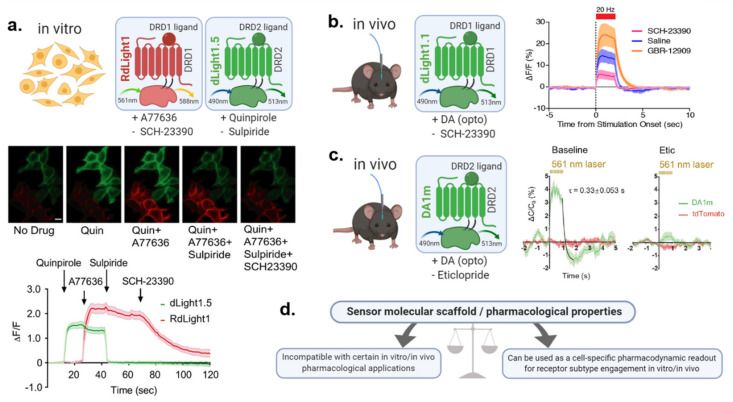
Molecular scaffold as a double-edged sword for pharmacology and drug discovery: (**a**) In vitro assays: [Top]: multiplex imaging of drug efficacy at distinct dopamine (DA) receptor subtypes in HEK293 cells expressing either RdLight1 (= DRD1 scaffold) or dLight1.5 (= DRD2 scaffold) in the same culture dish. [Middle]: representative images of HEK293 cells and their average fluorescent responses (dFF) to individual drugs. Scale bars, 10 μm. [Bottom]: Simultaneously measured fluorescence responses of both sensors during bath application of DRD1 drugs: A77636: 100 nM, SCH23390: 10 μM and DRD2 drugs: Quinpirole (Quin): 10 μM, Sulpiride: 400 nM [59]. (**b**,**c**) In vivo assays: photometry imaging of drug efficacy at DRD1 (**b**) and DRD2 (**c**) receptors in mice expressing dLight1.1 (= DRD1 scaffold) or GRAB-DA1m (= DRD2 scaffold) in the NAc or striatum following optogenetic (opto) stimulation of DA neurons in the midbrain and i.p. injection of drugs: SCH23390: 0.25 mg/kg, Eticlopride: 2 mg/kg (figures were reused with permission from References [58,60]). (**d**) Pharmacological properties of sensors as a result of their parent receptor scaffold as a double-edged sword. Etic: Eticlopride. “+”: agonists, “−“: antagonists. GBR-12909: DA reuptake inhibitor.

As outlined above, DA biosensors were generated using DRD1 (dLight1.1, 1.2 and 1.3a, 1.3b, YdLight1, RdLight1), DRD2 (dLight1.5 and all GRAB-DA sensors) or DRD4 (dLight1.4) as the molecular scaffold [58,59,60,61,101]. As a result, DA biosensors are sensitive to ligands that target their parent receptor. For instance, in vitro, we found that the dLight1.1 (=DRD1-scaffold) fluorescent response to DA was abolished by the DRD1 antagonists SKF-83566 and SCH-233990 but was unaffected by the DRD2 antagonists haloperidol and sulpiride [58]. In the absence of DA, the RdLight1 (= DRD1 scaffold) fluorescent response increased in response to the DRD1 agonist A77636 and was abolished by SCH-233990. Conversely, in the same assay, dLight1.5 (=DRD2 scaffold) fluorescence increased in response to the DRD2 agonist quinpirole and was abolished by the DRD2 antagonist sulpiride (Figure 6) [59]. Similarly, fluorescent responses to DA were abolished following application of the DRD2 antagonists haloperidol for all GRAB-DA sensors (=DRD2 scaffold) [60,61]. Some of these effects were confirmed at the in vivo level in mice: for instance, we found that optogenetically-evoked dLight1.1 fluorescence was blunted following ip injection of the DRD1 antagonist SCH-233990 [58] (Figure 6). Optogenetically-evoked GRAB-DA responses were also abolished following ip injection of the DRD2 antagonist eticlopride [60,61] (Figure 6). Thus, these findings indicate that DA biosensors appear to reflect the pharmacological properties of the parent DA receptors (i.e., DRD1 or DRD2) from which they were engineered. In effect, this is a double-edged sword for the end-user. 

On the one hand, this indicates that DA biosensors may be incompatible with certain in-vitro/in-vivo applications. End-users using DA biosensors as part of pharmacological, drug discovery or drug addiction research must carefully evaluate the affinity of their drug(s) of interest for the sensors. Full agonists/antagonists of DRD1/DRD2 will be of great concern but drugs such as DREADD agonists (e.g., clozapine, CNO [238,239]) or ketamine [240] also have medium to low affinity for DRD1 and/or DRD2, which may in turn affect fluorescent signals. If in doubt, the affinity of a drug for a given biosensor can be determined empirically in HEK293 cells using concentration-response curves, as shown in Figure 4 or otherwise in vivo as shown in Figure 6. An alternative plan is to use FSCV, a method which demonstrates lower cellular and molecular specificity but similar spatiotemporal resolution and which remains unaffected by DA ligands (see Section 2.1). Developing DA sensors with decreased affinity for DRD1/DRD2 ligands is an area of great interest.

On the other hand, the pharmacological profiles of DA biosensors may represent an opportunity to develop novel fluorescent assays for GPCR drug discovery. In vitro, ligand binding fluorescent assays could be developed using concentration-response curves, allowing to determine the relative affinity of a panel of drugs for one or several receptor subtypes. For instance, we showed that spectrally-orthogonal DRD1- (red) and DRD2-based (green) sensors can be expressed simultaneously to examine target engagement of DRD1 vs. DRD2 drugs (Figure 6) [59]. A similar principle can be applied to other neuromodulators by combining, for instance a green NE sensor (nLight1.3) and a red DA sensor (RdLight1) to screen DA/NE candidate ligands [59]. Moreover, these in vitro findings suggest that similar assays could be deployed in vivo, whereby red or green DA biosensors could be used separately or in combination to probe pharmacodynamic target engagement of receptor subtypes. In the future, this could represent an exciting in vivo functional assay for drug development, akin to PET imaging, that would harbor high spatiotemporal resolution, cell specificity and ability to perform screening in awake behaving animals. Future studies are warranted, however, to develop a broader palette of receptor subtype sensors with high specificity (e.g., DRD3, DRD4) and across a wider color palette.

## 6. Piloting Sensor Use in the Laboratory 

### 6.1. Sensor Validation at the Neuroanatomical Level

**Figure 7:**
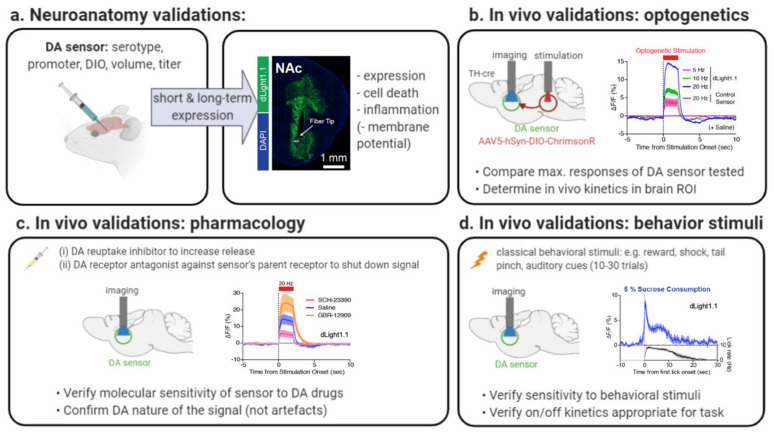
Piloting sensor use in the laboratory can follow these four steps: (**a**) First, optimal sensor expression in the region or cell of interest should be achieved by optimizing viral injections parameters, often based on protocols previously established in the laboratory for other viral vectors. After a minimum of 2 weeks of incubation, sensor expression can be evaluated as shown here for dLight1.1 in the nucleus accumbens (NAc) [58]. If experiments are expected to last more than 2 months, additional tests can be performed to verify that sensor expression does not induce cell death (e.g., caspase-3 staining) or inflammation (e.g., staining for reactive microglia or astrocyte markers), nor does it affect the basal properties of the cells of interest (membrane potential can be measured in slice physiology). (**b**) When piloting sensor use in a new brain region with possible sparse innervation, maximal sensor responses should be determined using optogenetic stimulation of dopamine (DA) neurons (e.g., measure NAc DA release using dLight1.1 after ventral tegmental area (VTA) DA neuron ChrimsonR stimulation [58], as shown here). Imaging of DA sensors should be performed using the imaging modality of choice and in vivo kinetics specific for this brain ROI determined by stimulating DA neurons at increasing frequencies (e.g., 5–20 Hz). (**c**) Pharmacological ligands can be used to validate DA sensors in vivo, e.g., (i) by using reuptake inhibitors e.g., GBR-12909 to increase release or (ii) by using DA receptor antagonists specific to the sensor’s parent receptor to decrease [58] (shown here) or abolish (see Reference [226]) fluorescent signals and verify that transients reflect DA release, e.g., using the DRD1 antagonist SCH-23390. (**d**) In a last step, sensors can be validated using classical behavioral events known to induce DA release in order to verify optimal detection of DA transients in response to physiological stimuli (e.g., measure dLight1.1 responses in the NAc after free sucrose consumption [58], as shown here). Note that native transients will be several-fold lower than after optogenetic stimulations, see Reference [58]). Users should also verify that sensor on/off- kinetics are compatible with the behavioral task of choice. Further information can be found in Reference [99].

To establish the best sensor for a new brain region, we recommend performing four simple pilot experiments (Figure 7); further experimental details can be found in our extended protocol [99]. First, the optimal construct parameters should be carefully chosen to allow good sensor expression in the cell/region of interest (Figure 7a). Parameters include AAV serotype (1 to 9 or DJ), AAV volume, AAV titer (high titer may lead to cell death) and promoter (e.g., CAG, hSyn, hEF1a, CBA). Often this will match parameters already used in the laboratory to express other molecules of interest. For instance, injection of 150 nL of an AAV9 expressing dLight1 under CAG or Syn promoter at 1 × 10^11^ viral genomes per milliliter (VG/mL) into the striatum and 2 weeks incubation time led to expression levels adequate for in vivo imaging with acceptable dFF values (~10–20%) for spontaneous peaks [99]. These values may vary from region to region, sensor to sensor or according to the in vitro or in vivo imaging modality (see Reference [99]). If prolonged imaging is required, for example, beyond 2 months, it is important to verify both the GPCR sensor expression levels and the health of the expressing cells. The latter can be performed for example by staining for DAPI to look at neuron numbers, neuroinflammatory markers like GFAP or cell death markers like caspase-3. Other possible validations may include examining cell firing properties in a slice electrophysiology preparation or evaluating the performance of sensor-expressing animals vs. control mice in a behavioral task.

### 6.2. Sensor Validation In Vivo Using Optogenetic Stimulation

In a second step, we recommend piloting sensor responses using optogenetic stimulation of afferent DA projections (e.g., of the VTA or SNc cell bodies), which will lead to maximal dFF responses and will allow to establish feasibility (Figure 7b). This can also allow to test the kinetics of the DA sensor, that is, at which maximal stimulation frequency can individual spikes be parsed out and what is the decay time in the brain region of interest. For instance, we found that in the NAc, dLight1.1 reliably tracks individual DA release events following VTA optogenetic stimulation of cell bodies up to 10 Hz [58]. Note that optogenetic stimulation and imaging can be done through different fibers [58] (Figure 7b) or through the same fiber or lens, as recently shown in Reference [226]. Optogenetic pilots should ideally be done in vivo to closely mimic the experimental conditions of the behavioral experiments. However, ex vivo slice experiments may also provide a useful tool to pilot multiple DA sensors at once and to screen sensor properties such as frequency dependency and sensitivity to DA ligands, as we have done previously [58]. In addition, if there is concern for strong NE innervation to the brain region, it may be useful to determine whether DA sensors in the brain region of interest are sensitive either to NE optogenetic stimulation or at least to drugs known to modulate NE release (as in Reference [61]) (note: it may be difficult to parse out indirect circuit effects). 

### 6.3. Sensor Validation In Vivo Using Pharmacology

Pharmacological ligands can also be used to validate DA sensors in vivo. On the one hand, reuptake inhibitors such as GBR-12909 can be used to increase release and verify sensitivity of the sensor to pharmacologically-induced modulation of release. For instance, we found that injection of 10 mg/kg GBR-12909 led to an increase in optogenetically-evoked DA release in the NAc [58]. Moreover, pharmacological antagonists specific to the sensor’s parent receptor (e.g., a DRD1 antagonist for the DRD1-based sensors dLight1.1, see Section 5.6) can be used to confirm that detected transients reflect DA release and not movement artefacts or electrical noise. For instance, injection of the DRD1 antagonist SCH-23390 at 0.25 mg/kg led to a reduction in NAc dLight1.1 fluorescent signal [58] while 10 mg/kg fully abolished it [226] (Figure 7c).

### 6.4. Sensor Validation In Vivo Using Behavioral Stimulation

In a last step, we recommend testing sensor responses using physiological stimulation such as behavioral stimuli known to affect DA release in the brain region of interest (Figure 7d). Detected transients can be several fold lower than after optogenetic stimulations, as shown in Reference [57] (see also Figure 1 and Section 4.3). For instance, sucrose rewards, reward-predicting auditory cues, electric shocks or aversive bitter solutions rapidly increase or decrease DA release in the NAc [58,60,102], dorsal raphe and central nucleus of the amygdala [38]; acute auditory stimuli like door-opening [113] increase DA release in the dorsal striatum, while tail pinches [23] and footshocks [98] elicit DA release in the mPFC. Use of trial repetitions (e.g., at least 10–30 presentations of the same stimuli) is particularly advantageous, because trial averaging allows even small or variable signals to be reliably detected. Moreover, kinetics should always be considered in relation to the behavioral task of interest. For example, we found that the t_1/2_ decay time of dLight1.1 sensor to be 100 ms but other sensors are slower (See Table 1). Thus, it is important to ensure that the intertrial interval (or time between two behavioral stimuli) is substantially higher than the t_1/2_ decay time to allow distinguishing responses to subsequent stimuli (see Section 5.3 for further details). Once the data has been collected, it can be analyzed similar to other sensors such as GCaMP.Detailed advice for data analysis can be found in other relevant publications [62,63,99,241,242].

## 7. Conclusions

Here we outlined the large palette of methods that currently exist to measure DA release, focusing a large part of our work on the recently developed DA biosensors, their advantages and limitations as well as their properties. We provided a detailed map of DA regional heterogeneity and argue that local brain DA levels should be accounted for when choosing a sensor based on its affinity. We then provide detailed guidelines for the end-user to optimize sensor choice according to other key sensor properties and experimental modalities. In studies that have already made use of DA biosensors, investigations have thus far primarily focused on the striatum and NAc, two regions with dense DA innervation. We are hopeful that the broad palette of sensors available and expected future developments in the field, will soon allow end-users to address the spatiotemporal dynamics of DA release during behavior in brain regions previously deemed inaccessible with classical methods. It is to be expected that further improvements in ligand affinities to the sensors as well as improvements in the dynamic range will significantly contribute to achieving this goal. Moreover, the more recently developed red-shifted DA sensor RdLight1 opens new doors to decipher neuromodulator dynamics in concert with multiple experimental modalities including optogenetic manipulations, multiplex imaging or two-color pharmacological drug screening. Future engineering efforts are expected to allow even more signals to be monitored simultaneously with enhanced sensitivity and specificity and across even broader imaging modalities. The modification of existing GPCR-based DA sensors could for example lead to the development of biosensors compatible with fluorescence life-time imaging, leading to the exciting possibility of measuring absolute DA concentrations in vivo in real-time (reviewed in Reference [118]).

## Figures and Tables

**Table 1:** The G protein coupled receptors (GPCR) dopamine biosensor toolbox and intrinsic properties.

Sensor	Molecular Scaffold	Affinity (K_d_/EC_50_)	Dynamic Range (dFF_max_)	Molecular Specificity vs. NE	On Kinetics: t_1/2_ Rise Time	Off Kinetics: t_1/2_ Decay Time	1-Photon Exc/Emis	Source
dLight1.1	DRD1	330 nM *	230% *	70-fold	10 ms **	100 ms **	490/517 nm	[58]
dLight1.2	DRD1	765 nM *	340% *	ND	9.5 ms **	90 ms **	490/517 nm	[58]
dLight1.3a	DRD1	2300 nM *	660% *	ND	ND	ND	ND	[58]
dLight1.3b	DRD1	1600 nM *	930% *	270-fold	ND	ND	ND	[58,101]
dLight1.4	DRD4	4 nM *	170% *	ND	ND	ND	ND	[58]
dLight1.5	DRD2	110 nM *	180% *	ND	ND	ND	ND	[58]
RdLight1 (red)	DRD1	860 nM *	250% *	60-fold	14 ms **	400 ms **	560/588 nm	[59]
YdLight1 (yellow)	DRD1	1630 nM *	310% *	ND	ND	ND	514/525 nm	[59]
GRAB-DA1m	DRD2	130 nM *	90% *	10-fold	60 ms *	710 ms *	490/510 nm	[60]
GRAB-DA1h	DRD2	10 nM *	90% *	10-fold	140 ms *	2520 ms *	490/510 nm	[60]
GRAB-DA2m	DRD2	90 nM *	340% *	22-fold	40 ms *	1300 ms *	ND	[61] †
GRAB-DA2h	DRD2	7 nM *	280% *	15-fold	50 ms *	7300 ms *	500/520 nm	[61] †
GRAB-rDA1m (red-shifted)	DRD2	95 nM *	150% *	15-fold	80 ms *	770 ms *	565/595 nm	[61] †
GRAB-rDA1h (red-shifted)	DRD2	4 nM *	100% *	10-fold	60 ms *	2150 ms *	565/595 nm	[61] †

DRD1,2,4: dopamine receptor 1,2 or 4 subtype; Exc: excitation wavelength; Emis: emission wavelength; dFF_max_: maximal increase in fluorescence between ligand-free and ligand saturated states; ND: not determined; * estimated in HEK-293 cells following bath or puff ligand application; ** estimated in brain slices following 1 electrical pulse; † preprint (not peer-reviewed) at the time of this publication.

## Supplementary Materials

The following are available online at https://www.mdpi.com/1422-0067/21/21/8048/s1, Table S1: Microdialysis, Table S2: DA Sensor Applications.

## Abbreviations

AAV	Adeno-associated virus
Amy	Amygdala
AP	Action potential
Au1	Primary auditory cortex
BNST	Bed nucleus of the stria terminalis
CAG	Human cytomegalovirus promoter
cAMP	Cyclic adenosine monophosphate
CNiFERs	Cell-based neurotransmitter fluorescent engineered reporters
CNO	Clozapine-n-oxide
cpFP	Circularly-permuted fluorescent protein
cpGFP	Circularly-permuted green fluorescent protein
cpmApple	Circularly-permuted mApple (fluorescent protein)
CPu	Caudate-Putamen (= striatum)
CRB	Cerebellum
DA	Dopamine
dFF	Normalized fluorescent response (ΔF/F)
DR	Dorsal raphe nuclei
DRD1-5	Genes encoding D1-D5 dopamine receptors
EC50	Half maximal effective concentration
Emis	Emission wavelength
ENT	Enthorhinal cortex
Exc	Excitation wavelength
FFNs	False fluorescent neurotransmitters
FRET	Förster resonance energy transfer
FSCV	Fast-scan cyclic voltammetry
GECIs	Genetically-encoded calcium indicators
GFAP	Glial fibrillary acidic protein
GPCR	G protein-coupled receptor
GPe	Globus pallidus externus
GRAB	GPCR-activation based sensors
GTP	Guanosine triphosphate
HEK-293	Human embryonic kidney cell line
HPC	Hippocampal formation
HPLC	High-performance liquid chromatography
hSyn	Human synapsin promoter
Hyp	Hypothalamus
Kd	Dissociation constant
LS	Lateral septum
M1	Primary motor cortex
M2	Secondary motor cortex
mPFC	Medial prefrontal cortex
MS	Medial septum
NAc	Nucleus accumbens
ND	Not determined
NE	Norepinephrine
OT	Olfactory tubercle
PBP	Periplasmic Binding Protein
PVT	Paraventricular thalamus
ROI	Region of Interest
SN	Substantia nigra
SNc	Subtantia nigra pars compacta
SNR	Signal-to-noise ratio
SWCNT	Single-walled carbon nanotubes
TeA	Temporal association cortex
TEV	Tobacco Etch Virus peptide sequence
TIRF	Total internal reflection microscopy
TRE	Tetracycline response element
tTA	Tetracycline-controlled transactivator
VP	Ventral pallidum
VMAT2	Vesicular monoamine transporter 2
VTA	Ventral Tegmental Area
